# Gastric cancer-derived exosomal miR-519a-3p promotes liver metastasis by inducing intrahepatic M2-like macrophage-mediated angiogenesis

**DOI:** 10.1186/s13046-022-02499-8

**Published:** 2022-10-10

**Authors:** Shengkui Qiu, Li Xie, Chen Lu, Chao Gu, Yiwen Xia, Jialun Lv, Zhe Xuan, Lang Fang, Jing Yang, Lu Zhang, Zheng Li, Weizhi Wang, Hao Xu, Bowen Li, Zekuan Xu

**Affiliations:** 1 Department of General Surgery, The First Affiliated Hospital of Nanjing Medical University, 300 Guangzhou Road, Nanjing, 210029 Jiangsu Province China; 2 Department of General Surgery, The First People’s Hospital of Nantong, The Second Affiliated Hospital of Nantong University, Nantong, 226001 Jiangsu Province China; 3 Jiangsu Key Lab of Cancer Biomarkers, Prevention and Treatment, Collaborative Innovation Center for Cancer Personalized Medicine, Nanjing Medical University, Nanjing, 211166 Jiangsu Province China

**Keywords:** Gastric cancer liver metastasis, Exosomes, miR-519a-3p, M2-like polarization, Angiogenesis

## Abstract

**Background:**

Liver metastasis (LM) is a major obstacle to the prognosis of gastric cancer (GC) patients, but the molecular mechanism underlying gastric cancer liver metastasis (GC-LM) remains unknown. Exosomes have been identified as an important mediator of communication between tumor cells and the microenvironment. Therefore, we sought to investigate the effects of primary GC cells on the liver microenvironment and the role of exosomal microRNAs (exo-miRNA) in GC-LM.

**Methods:**

Sequential differential centrifugation, transmission electron microscopy and NanoSight analysis were used to extract and characterize exosomes. MicroRNA sequencing in GC-derived exosomes and mRNA sequencing in PMA-treated THP-1 cells were used to identify differentially expressed miRNAs in exosomes and the functional targets of exosomal miR-519a-3p (exo-miR-519a-3p) in macrophages, respectively. Tracing and internalization of exosomes and transfer of exo-miR-519a-3p were observed by immunofluorescence. Tubule formation assays, aortic ring assays, and exosome-educated GC-LM model were used to investigate the roles of GC-derived exosomes and exo-miR-519a-3p in angiogenesis and GC-LM. Luciferase reporter assay, qRT-PCR, Western blot, ELISA, flow cytometry and immunofluorescence were used to investigate the regulatory mechanism of exo-miR-519a-3p at GC-LM.

**Results:**

The expression level of miR-519a-3p in serum exosomes was significantly higher in GC-LM patients than in patients without LM, and high expression of exo-miR-519a-3p indicates a worse prognosis. GC-derived exosomes are mainly accumulated in the liver and internalized by intrahepatic macrophages. Mechanistically, exo-miR-519a-3p activates the MAPK/ERK pathway by targeting DUSP2, thereby causing M2-like polarization of macrophages. M2-like polarized macrophages accelerate GC-LM by inducing angiogenesis and promoting intrahepatic premetastatic niche formation.

**Conclusions:**

Our results indicate that exo-miR-519a-3p plays a critical role in mediating crosstalk between primary GC cells and intrahepatic macrophages and is a potential therapeutic target for GC-LM.

**Supplementary Information:**

The online version contains supplementary material available at 10.1186/s13046-022-02499-8.

## Introduction

Distant metastases are a determining factor for poor prognosis in patients with gastric cancer (GC) [1]. Much of the blood from the gastrointestinal tract flows to the liver via the portal vein, and hematologic metastasis is the major route for the spread of tumor cells to distant sites, making the liver the most important target organ for GC metastasis. It is reported that about 4–14% of GC patients suffer from liver metastasis (LM) [2]. There are relatively few treatment options for gastric cancer with liver metastases (GC-LM), and chemotherapy remains the mainstay, albeit with limited efficacy [3]. Therefore, there is an urgent need to explore the underlying mechanisms to identify the critical therapeutic targets, develop effective treatment strategies, and ultimately improve the prognosis of patients with GC-LM.

Exosomes, extracellular membrane vesicles approximately 30–150 nm in diameter [4, 5], have been shown to be involved in the development of many malignancies [6–8]. Exosomes contain abundant proteins, dsDNA, ssDNA, mRNAs and miRNAs [9], which are delivered to specific target organs where they are involved in remodeling the tumor microenvironment [10]. MicroRNA (miRNA) is a small non-coding RNA with a length of 17–24 nt that regulates the expression of target genes by binding to their 3'UTR regions, thereby exerting a variety of biological functions [11]. It has been found that miRNAs account for about 43% of RNA in exosomes and play an important role in the bioregulatory functions of exosomes [9]. Exosomal miRNAs (exo-miRNAs) have also been shown to be involved in organ-specific metastasis of various cancers, including lung, breast, pancreatic, and melanoma cancers, by remodeling the target organ microenvironment [12–15]. However, studies on the role and mechanisms of exosomal miRNAs in GC-LM are still relatively lacking.

Recently, the role and significance of the pre-metastatic ecological niche in metastasis has received increasing attention. The formation of the pre-metastatic niche is a complex process in which components derived from the primary tumor impact distant organs and undergo a series of molecular and cellular changes to create a supportive and receptive tissue microenvironment for the colonization of circulating tumor cells, thereby facilitating tumor metastasis [16]. Primary tumor components, cells mobilized from the bone marrow by the tumor, and the local host stromal microenvironment are the three key factors involved in the formation of the premetastatic niche [17]. As an important component of the local stromal microenvironment, macrophages are crucial in pre-metastatic niche formation. Liver macrophages, consisting of self-sustaining liver-resident Kupffer cells (KCs) and recruited inflammatory monocyte-derived macrophages (Mo-Mfs), are key participants in maintaining liver homeostatic function and responding to pathological injury [18]. It has been reported that macrophages can be converted into different phenotypes in response to the dynamically changing tumor microenvironment, usually classified as classically activated phenotype (M1) and alternatively activated phenotype (M2-like) [19]. Increased vascular permeability and neo-angiogenesis are key steps in the establishment of the pre-metastatic niche [17]. M2-like macrophages have been found to secrete many potent pro-angiogenic cytokines, growth factors, and angiogenic regulatory enzymes [20], which trigger the angiogenic switch and promote metastasis formation. Thus, when macrophages in the receiving organ are induced to M2-like polarization, the "soil" suitable for circulating tumor cells becomes more fertile and the time to metastasis is dramatically shortened.

In this study, we aimed to elucidate the regulatory mechanisms underlying GC-derived exo-miRNAs in GC-LM. We examined the expression pattern of miR-519a-3p in GC-LM and evaluated whether exo-miR-519a-3p promotes premetastatic niche formation by inducing M2-like polarization of intrahepatic macrophages. We hope that our results can help identify a novel biomarker specific to GC-LM and provide new ideas for potential therapeutic strategies against LM.

## Methods

### Patient samples

Tissue samples were collected from GC patients diagnosed by gastroscopy, puncture pathology and CT at First Affiliated Hospital of Nanjing Medical University (including 30 patients with stage III and 30 GC patients with liver metastases). The tissue samples mainly included gastric cancer tissue, normal paracancer tissue, liver metastasis tissue and normal liver tissue. The fresh tissue was frozen and stored in liquid nitrogen until use. Blood samples were collected preoperatively and on the seventh postoperative day from patients who underwent surgical treatment. In addition, healthy blood samples were collected from 15 volunteers without malignancy or acute/chronic disease. All blood samples were centrifuged at 2,500 g for 10 min to extract serum and then stored at -80 °C until use. Prior approval was obtained from the Nanjing Medical University Ethics Committee, and informed consent was obtained from each patient.

### Cell culture

The human gastric epithelial cell line GES -1 and GC cell lines (validated by Short Tandem Repeat DNA fingerprinting) were purchased from the Cell Bank of the Type Culture Collection of the Chinese Academy. MKN45, MKN45-HL, HGC-27, and NCI-N87 were cultured in RPMI-1640 (w/o Hepes). KATOIII and AGS were cultured in Dulbecco's Modified Eagle's medium (DME H-21 4.5 g/liter glucose) and Nutrient Mixture F-12 K, respectively. To the nutrient medium, 10% fetal bovine serum (Gibco, USA), 1% antibiotics (HyClone, USA), and 0.1% mycoplasma antibiotics (Invitrogen, USA) were added. All cells were proliferated in an environmental incubator (humidified atmosphere with 5% CO_2_, 37 °C).

### Establishment of highly liver metastatic potential gastric cancer cells

Gastric cancer cells with high liver metastatic potential (MKN45-HL) were successfully established in our previous study [21]. In brief, 2 million MKN45 cells stably transfected with a double-labeled empty GFP-Flag lentivirus (Shanghai Gene Pharma) were injected into the hepatic portal vein of 6-week-old female BALB/c nude mice. 3 weeks later, the mice with liver metastases were sacrificed, and the livers were harvested and then processed into single cell suspensions. The labeled GC cells were sorted out from the single cell suspensions by flow cytometry (FC). The extracted GFP-positive MKN45 cells were then re-injected into the hepatic portal vein to obtain cells with a greater capacity for liver metastasis. After repeating the above steps three times, the flow cytometrically sorted GFP-positive cells were classified as GC cells with high liver metastatic potential.

### Exosomes isolation and identification

MKN45 and MKN45-HL cells were cultured in normal medium until they were 80% confluent. Then the medium was replaced with exosome-depleted medium (RPMI1640 containing 10% exosome-depleted FBS). Two days later, the conditioned medium (approximately 15 ml) was collected from each dish and subjected to polymerization precipitation, ultrafiltration, and ultracentrifugation to harvest exosomes. Briefly, the conditioned medium was successively centrifuged at 1,00 × g for 10 min, 2,000 × g for 10 min, and 18,000 × g for 30 min to remove cell fragments, cell debris, and large extracellular vesicles (lEVs). The supernatant was then concentrated using an ultrafiltration tube (Merck Millipore) to < 3 mL (4,000 × g, 4 °C) to separate the exosome fraction from the soluble exosome fraction. Exosomes were then purified according to the experimental protocol of Total Exosome Isolation Reagent (Invitrogen; 4,478,359). The pellet was resuspended in particle-free PBS or stored at -80 °C in the refrigerator for subsequent use. For exosome purification from plasma, blood samples must be successively centrifuged at 150 g for 15 min and 1,200 g for 15 min to obtain PPP (platelet-poor plasma). The separation and concentration of exosomes in PPP were then similar to that of the cell-conditioned medium.

The NanoSight LM10 system (Malvern Instruments, UK) was used to analyze the amount and concentration of exosomes. Exosomes were loaded into the sample chamber of the LM10 unit, and the fast video recording and particle tracking software measured the speed of Brownian motion with the same detection threshold. The exosome concentration was calculated as an average value. In addition, the morphology of exosomes was determined by transmission electron microscopy (TEM, JEOL, Japan). The expression of the exosome markers CD81, TSG101, and the lEVs marker calnexin was detected by Western blot after measuring the protein concentration of exosomes using the BCA protein assay kit (ThermoFisher Scientific, USA). Information on antibodies detecting the above markers is provided in Table S1 in the supplemental material.

### Exosome labeling and tracking

Exosome tracking assays were performed as previously described [21]. A total of 10 μg exosomes were first mixed with Diluent C buffer containing 4 μl PKH26/67 dye (Sigma, USA) for 5 min and then incubated with 1% BSA solution at room temperature. For exosome tracking assays in vivo, 10 μg PKH26-labelled exosomes resuspended in 100 μl PBS were injected into BALB/c nude mice (6 weeks old) via the tail vein. Twenty-four hours later, the mice were sacrificed, and the brain, lung, liver, spleen, and bone tissues were harvested for in vivo imaging. To determine whether exosomes were taken up by macrophages in vitro, 10 µg of PKH-26/67-labelled exosomes were resuspended in PBS solution and incubated with PMA-treated THP-1 cells at 37℃ for 4 h. THP-1 cells were then stained with DAPI and observed by confocal laser microscopy.

### Small RNA sequencing analysis of exosomes (exo-miRNA-seq)

Exosomes were extracted from the conditioned medium of MKN45 and MKN45- HL cells. Total RNAs in exosomes were extracted after exosome characterization. Small RNA sequencing was performed in an Illumina HiSeq 2500 system from Huayin Health Co (Guangzhou, China). Briefly, 35 ng of exosome RNA was converted to cDNA after ligation with RNA 3' and 5' adapters and amplified with Illumina primers. Raw data were generated and then subjected to initial quality control to weed out sequences less than 16 nt in length. The miRBase database was used as a reference genome for human miRNA sequences. Raw data normalized by TPM were analyzed using the edgeR package (Bioconductor Software).

### RNA extraction and qRT-PCR

TRIzol reagent (Technologies, USA) was used to extract total RNA from GC cells and tissues. The miRNA and mRNA were transcribed into cDNA using the New Poly(A) Tailing Kit (ThermoFisher, USA) and PrimeScript RT Master Mix Kit (TaKaRa, Japan), respectively, according to the manufacturer's protocol. The above cDNAs were then amplified using the FastStart Universal SYBR Green Master Kit (Roche, Mannheim, Germany) and detected using the ABI PRISM 7900HT Sequence Detection System (Applied Biosystems, Waltham, MA, USA). The relative expression of miRNA and mRNA was normalized to U6 and β-actin, respectively. Primer information is shown in Table S2 in the supplemental material.

### Protein extraction and Western blot

RIPA Lysis buffer (Beyotime, Shanghai, China) supplemented with protease and phosphatase inhibitors (NCM Biotech, Suzhou, China) was used to extract proteins from cells and tissues. SDS-PADE Protein buffer (Beyotime, Shanghai, China) was added to the lysate, and proteins were then denatured in a water bath at 100 °C for 5 min. Protein samples were transferred to polyvinylidene difluoride membranes (Thermo Fisher, MA, USA) after separation by SDS–polyacrylamide gel electrophoresis. The above membranes were blocked with 5% fast blocking buffer (Beyotime, Shanghai, China) for 45 min and then incubated with primary antibodies overnight at 4 °C in a refrigerator and then immunoblotted with secondary antibodies for 2 h. Finally, images of the blots on the membranes were acquired using a Tanon-4600 Imaging System (Biotanon, China). Information on the primary and secondary antibodies used in the Western blot is given in Table S1 in the supplemental material.

### Flow cytometry

To detect CD206 + macrophages, PMA-treated THP-1 cells were first harvested and fixed overnight in 1% paraformaldehyde (PFA) at 4 °C. They were then resuspended in flow cytometry buffer (1 × PBS buffer containing 1% FSA) and stained with anti-CD206 (BD Biosciences, USA) for 30 min at room temperature.

### Cytokine assay

To investigate the effect of exosomes on the expression of TGF-β, VEGFA, and VEGFD in THP-1 cells, enzyme-linked immunosorbent assay (ELISA) was performed using the TGF beta-1 Human ELISA kit, the vascular endothelial growth factor A Human ELISA kit, and the VEGF-D (FIGF) Human ELISA kit (Thermo Fisher Scientific, USA). Absorbance was measured at 490 nm using a spectrophotometer (Thermo Fisher Scientific, USA) within 20 min after completion of the reaction. Cytokine concentrations were scaled according to the standard curve.

### Fluorescence in situ hybridization (FISH) and Immunohistochemistry (IHC)

Paraffin-embedded tissue blocks were cut into 2.5 μm sections and spread on glass slides. FISH was performed using miR-519a-3p detection probes (RiboBio, Guangzhou, China) and a fluorescence in situ hybridization kit (FISH) (Bosterbio, USA) according to the manufacturer's protocol. For immunohistochemistry (IHC), endogenous peroxidase activity was first blocked with 3% hydrogen peroxide, and then the sections were incubated with the primary antibody overnight at 4 °C. The next day, the sections were incubated with secondary antibody for 1 h at room temperature and then stained with 3,3-diaminobenzidine solution and hematoxylin. Antibody information is provided in Table S1 in the supplemental material.

### Immunofluorescence (IF)

For histological analysis, liver tissues embedded in paraffin were first baked at 60 °C for 1 h and then deparaffinized with xylene and anhydrous ethanol. The tissues were sequentially treated with Triton X-100, enhanced citrate antigen retrieval solution, and Enhanced Endogenous Peroxidase Blocking Buffer (Beyotime Biotechnology, China) to break the membrane, repair the antigen, and eliminate endogenous peroxidation. After blocking with QuickBlock™ for one hour, the tissues were incubated overnight at 4 °C with the primary antibody. The next day, tissues were incubated with fluorescently labeled secondary antibody for 2.5 h before staining nuclei with DAPI. Fluorescence images were acquired and analyzed using a Thunder Imager (LEICA, Germany) fast, high-resolution, inverted fluorescence imaging system.

### Small interfering RNA, miRNA inhibitor/mimics, and lentivirus transfection

The miR-519a-3p inhibitor/mimics or negative controls were purchased from GenePharma Co. Lentivirus vectors expressing miR-519a-3p and suppressing miR-519a-3p were designed and manufactured by ViGene Biosciences Co. In rescue experiments, PMA-treated THP-1 cells were transfected with miR-519a-3p mimics together with DUSP2 overexpression plasmid (ViGene Biosciences Co). Sequence information is shown in Table S2 in the supplemental material.

### mRNA sequencing (mRNA-seq)

PMA-treated THP-1 cells were transfected with miR-519a-3p inhibitor and inhibitor-NC before total RNA was extracted with TRIzol (Life Technologies, USA). After quality control with Nanodrop 2000, total RNA was temporarily stored at -80 °C, and high-throughput mRNA sequencing was performed on the BGIseq 500 platform (BGI-Shenzhen, China).

### Dual-luciferase reporter assay

The wild-type or mutant 3'UTR of DUSP2 was constructed into dual luciferase reporters using pmirGLO vector (Kinkairui, China), and 60 nM miR-519a-3p mimics or negative control (GenePharma, China) was cotransfected with 1 μg of the wild-type or mutant luciferase reporter plasmids in HEK293T cells. Relative luciferase activity was measured using a dual luciferase reporter assay kit according to the manufacturer's protocol (Promega, USA). Fluorescence signal was detected using the GloMax ® 20/20 instrument (Promega, USA).

### Migration assay

Before performing cell function assays, an in vitro co-culture system was established. In brief, HUVECs (human umbilical vein endothelial cells) suspended in 200 ml medium were seeded in the upper chamber (diameter = 0.4 μm; Corning, USA), and exosome-incubated or plasmid-transfected THP-1 cells in 800 μl medium containing 10% FBS were added to the lower chamber. After 48 h of coculture, HUVECs were separated from the upper chamber and prepared for subsequent experiments. Transwell chambers (diameter = 8 μm; Corning, USA) were used to study the migration ability of HUVECs. After 24 h of incubation, cells were stained with 0.1% crystal violet for 30 min and 6 random 100 × fields of view were selected to count the number of migrating cells.

### Tube formation assay

Matrigel (Corning, USA) was first thawed at 4 °C and spread into 96-well plates with 50 μl in each well and rested at 37 °C for 30 min to form a gel. The supernatant of PMA-treated THP-1 cells incubated with different exosomes was collected, and 1 × 10^4^ HUVECs were suspended in 100 μl of the above supernatant. After 6-h incubation, tube formation was photographed with a digital camera system (Olympus, Japan), and the total length and number of branches of the tubes were analyzed with Image J software.

### Aortic ring assay

The thoracic aorta was obtained from 6- to 8-week-old BALB/c nude mice in a sterile environment. The aortic rings (cut into 5-mm-long lengths) were placed in wells coated with Matrigel and incubated at 37 °C for 30 min to allow the Matrigel to polymerize. DMEM/F12-K medium (Gibco, USA) containing 10% fetal bovine serum and 1% antibiotics (HyClone, USA) supplemented with 10 ng/ml VEGF and 25 μg/ml heparin were then added to the wells. Twenty-four hours later, aortic rings were cultured with conditioned medium from PMA-treated THP-1 cells incubated with GC cells-derived exosomes or transfected with miR-519a-3p mimics, miR-519a-3p inhibitors, or DUSP2 overexpression plasmids. After one week of culture, vessel growth was quantified by microscopic counting of all buds on each aortic ring.

### Animal models

The animal experimental protocols of this study were reviewed and approved by the Animal Ethics Committee of Nanjing Medical University. All mice were purchased from the Laboratory Animal Centre of Nanjing Medical University and raised under pathogen-free conditions. The exosome-educated mouse models were established as described previously. In brief, mice were first randomly divided into several groups according to the requirements of the experimental design. 10 μg of exosomes from differentially treated MKN45 and MKN45-HL cells were resuspended in 150 μl PBS and then injected via the tail vein of the mice. This injection of exosomes via the tail vein was repeated every 2 days for 2 weeks, which we termed exosome education. At the end of exosome education, 2 million MKN45 cells transfected with luciferase lentivirus were injected into the mice via the spleen. Three weeks after spleen injection, the mice underwent in vivo imaging (Calliper Life Sciences, Hopkinton, MA, USA) and were subsequently sacrificed to harvest their livers for photographs and H&E staining.

For the subcutaneous tumor mouse model, 5-week-old female mice were randomly divided into 4 groups, and then 1 × 10^7^ GC cells stably knockdown- or overexpressed with miR-519a-3p were suspended in 150 μl PBS and then subcutaneously injected into the axilla of the forelimb of each mouse. The volume (V = length × width^2^ × 0.5) of xenograft tumors was measured each week. The mice were sacrificed four weeks later, and the subcutaneous tumors were harvested, weighed, and photographed.

### Extraction of mouse Kupffer cell

To extract intrahepatic macrophages from mice, mice educated with exosomes were sacrificed and hepatocyte-monocyte suspensions were prepared. Then, 2 mL liver monocyte suspension, 50% Percoll (Sigma, USA), 25% Percoll, and 1 mL PBS were slowly added to a 10 mL centrifuge tube and centrifuged at 1600 rpm for 25 min at 4 °C. After centrifugation, the white cell mass was collected between 50% Percoll and 25% Percoll in a new tube, and 10 mL of PBS was added. The tubes were mixed and centrifuged at 200 rpm for 7 min, and then the supernatant was discarded. Cells were inoculated into 10 cm cell cultures or total RNA, or protein was extracted directly from the cells for subsequent assays.

### Statistical analysis

All experiments were repeated more than 3 times. Quantitative data were expressed as mean ± standard deviation. Student's *t* test was performed to analyze the statistical difference between two groups, and analysis of variance (ANOVA) was applied to evaluate the differences between multiple groups. Kaplan–Meier method and log-rank test were used to analyze overall survival. The *p*-value < 0.05 was defined as statistically significant. For exosome miRNA-seq and mRNA-seq, the corrected *p*-value < 0.05 was defined as statistically significant.

## Results

### GC-derived exosomes promote liver metastasis

**Fig. 1:**
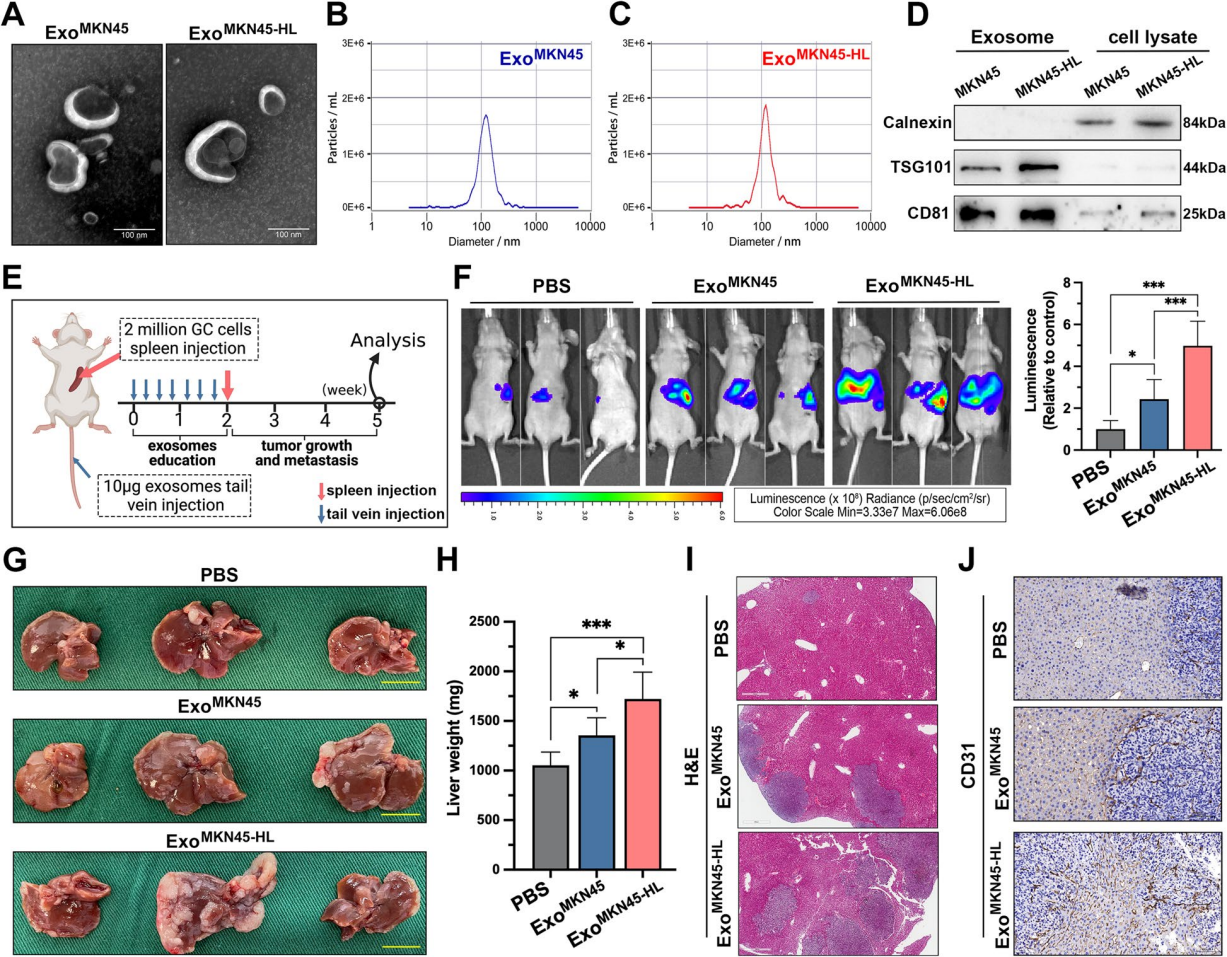
GC-derived exosomes promote liver metastasis. **A**. Representative images of exosomes from MKN45 and MKN45-HL cells at TEM, Scale bar = 100 nm. **B**, **C**. Purified MKN45 and MKN45-HL exosomes were analyzed by NanoSight. **D**. Western blot analysis of exosome markers (TSG101 and CD81) in exosomes and lysates from MKN45 and MKN45-HL cells. **E**. Schematic representation of the establishment process of the exosome-educated GC-LM model. **F**. Representative in vivo imaging system (IVIS) results of mice injected with luciferase-labeled MKN45 cells into the spleen after educated with PBS, MKN45 exosomes, or MIN45-HL exosomes, respectively. Results of quantified values of bioluminescence imaging signals are expressed as mean ± standard deviation (*n* = 6). **G**-**I**. Effect of GC-derived exosomes on liver metastasis in mice. The representative photographs of liver metastasis, liver weight, and **H**&**E** staining were shown. The scale bars are 1.0 cm and 500 μm, respectively. **J**. Representative images of CD31 immunohistochemical staining of LM tissues from mice with exosome education, Scale bar = 100 μm. Data are shown as mean ± standard deviation of 3 independent experiments, and statistical significance was determined using one-way ANOVA test (*P < 0.05, **P < 0.01, ***P < 0.001)

To investigate the mechanisms underlying GC-LM, a previously established GC cell line (MKN45-HL) with increased migration and invasion ability and high liver metastatic potential was used for our study [21]. Exosomes were first isolated from the supernatant of MKN45 and MKN45-HL cells by ultracentrifugation and then characterized and quantified by electron microscopy and nanoparticle tracking analysis (NTA). As shown in Fig. 1A, the typical cup-shaped and double-membrane-structured particles with diameters ranging from 30 to 150 nm were observed by electron microscopy, and NTA also revealed that exosomes from both cell lines were slightly larger than 100 nm (Fig. 1B, C). In addition, the purity of exosomes derived from MKN45 and MKN45-HL was assessed by analysis of exosome markers (TSG101 and CD81) and confirmation of the absence of endoplasmic reticulum (calnexin) (Fig. 1D). To investigate whether GC cell-derived exosomes could promote GC-LM, an exosome-educated GC-LM model were constructed (Fig. 1E). We intravenously injected mice with PBS, MKN45 exosomes, and MKN45-HL exosomes in the tail vein, followed by luciferase-labeled MKN45 cells in the spleen. After three weeks, in vivo imaging was performed, and the results showed that GC-derived exosomes significantly increased the fluorescence intensity in mouse liver compared with the PBS group, and the strongest fluorescence signal was observed in MKN45-HL-derived exosome-educated mice (Fig. 1F). Mice were then sacrificed, livers weighed and subjected to H&E staining. The results showed that GC-derived exosomes significantly increased the size and number of liver metastases, especially MKN45-HL-derived exosomes, compared with the PBS group (Fig. 1G-I). Furthermore, immunohistochemical staining of CD31 revealed that education with GC-derived exosomes resulted in significantly more disorganized blood vessels in and around liver metastases compared with PBS (Fig. 1J). Thus, these results demonstrated that GC-derived exosomes play an important regulatory role in GC-LM.

### GC-derived exosomes promote M2-like polarization of intrahepatic macrophages

**Fig. 2:**
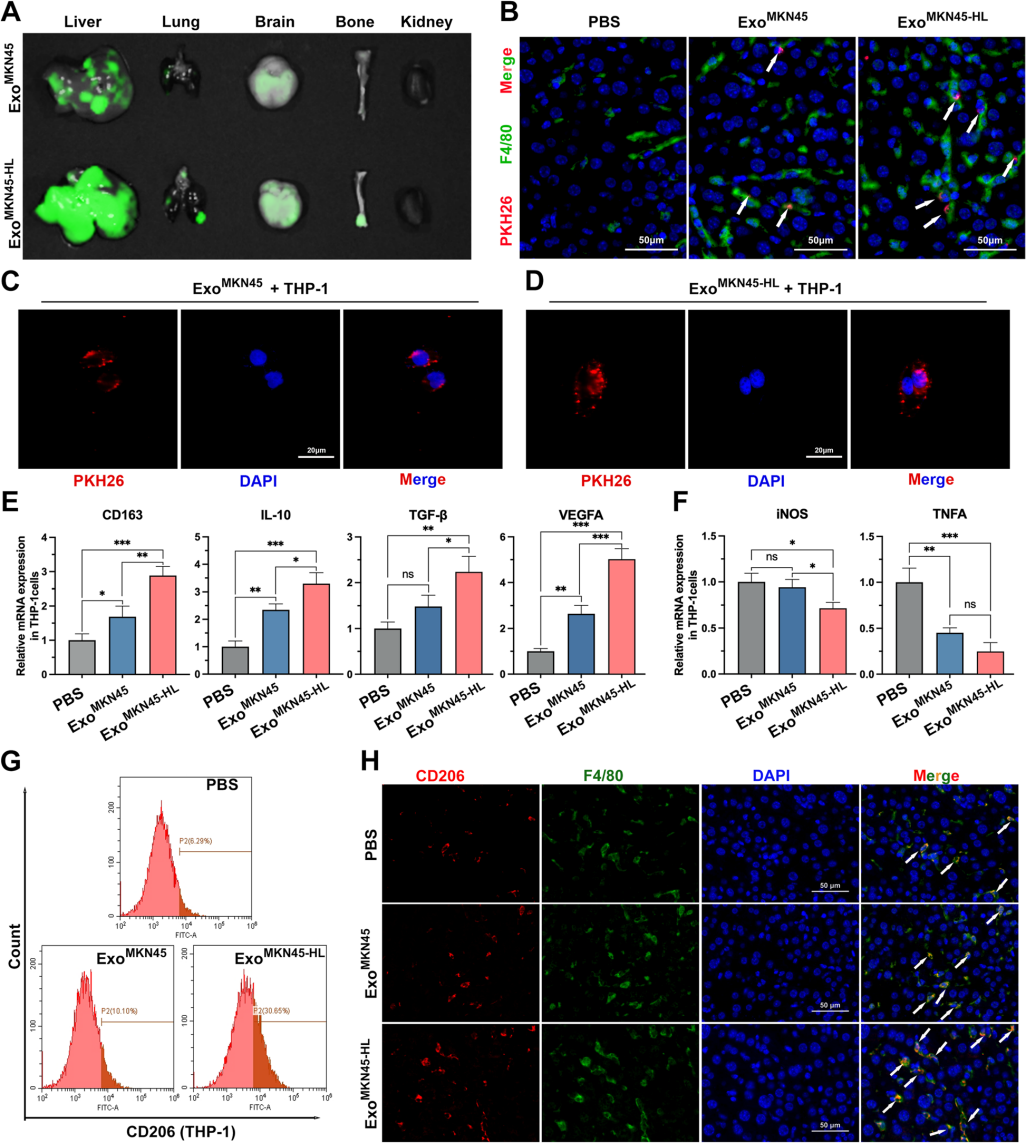
GC-derived exosomes promote M2-like polarization of intrahepatic macrophages. **A**. Representative fluorescence images of mouse liver, lung, brain, bone, and kidney tissues after injection of PKH26-labelled exosomes from MKN45 and MKN45-HL cells into the tail vein. **B**. Representative immunofluorescence showing colocalization between exosomes and macrophages (F4/80) in mouse liver after tail vein injection of PKH26-labeled exosomes. Scale bar = 50 μm. **C**, **D**. Representative immunofluorescence image showing internalization of PKH26-labeled MKN45/MKN45-HL-derived exosomes (red) by PMA-treated THP-1 cells. Scale bar = 20 μm. **E**, **F**. The expression of typical M2 markers (CD163, IL10, TGF-β, and VEGFA) and M1 markers (iNOS and TNFA) in PMA-treated THP-1 cells incubated with MKN45/MKN45-HL-derived exosomes or PBS (control) were examined by qRT-PCR. **G**. The effect of GC cell-derived exosomes on the expression of CD206 (M2 marker) was detected by flow cytometry. **H**. Immunostaining of CD206 (M2 marker) and F4/80 (macrophage marker) in livers of mice educated with PBS, MKN45 exosomes or MKN45-HL exosomes. Scale bar = 50 μm. Data are shown as mean ± standard deviation of 3 independent experiments, and statistical significance was determined using one-way ANOVA test (*P < 0.05, **P < 0.01, ***P < 0.001)

To investigate the organs susceptible to GC-derived exosomes, fluorescently labeled exosomes from MKN45 and MKN45-HL cells were injected into mice via the tail vein. Analysis of fluorescence intensity in each organ revealed that GC-derived exosomes were significantly more enriched in the liver than in other organs in all cases. In addition, exosomes derived from MKN45-HL cells were more prone to internalization by the liver than exosomes from MKN45 cells (Fig. 2A and Supplementary Fig. S1A). We next examined the predominant cells most likely to take up tumor exosomes in the liver. As shown in Fig. 2B, immunofluorescence staining of liver tissue revealed that both MKN45-HL- and MKN45-derived exosomes (PKH26-labeled) were colocalized with intrahepatic macrophages (F4/80 +). Subsequently, human THP-1 monocytes treated with 12-myristate-13-acetate (PMA) were used to mimic macrophages. The stimulated cells exhibited markedly adherent morphology and highly expressed the recognized macrophage marker CD68 (Supplementary Fig. S1B). Tumor-derived exosomes were then co-cultured with PMA-treated THP-1 cells, and the results showed that both MKN45-HL- and MKN45-derived exosomes were internalized by macrophages (Fig. 2C, D). Macrophages have been found to transform into classically activated phenotypes (M1) and alternatively activated phenotypes (M2-like) as a consequence of changes in the microenvironment, and M2-like macrophages play an important role in tumor development and metastasis [22–24]. Our qRT-PCR results revealed that MKN45-HL exosomes significantly increased the expression of CD163, IL-10, TGF-β, and VEGFA (M2 macrophage markers) and significantly decreased the expression of iNOS and TNFA (M1 macrophage markers) in PMA-treated THP-1 cells compared with PBS and MKN45 exosomes (Fig. 2E, F). Subsequently, the expression of CD206 in PMA-treated THP-1 cells was detected by flow cytometry, and the results indicated that MKN45-HL-exo significantly promoted the expression of CD206 (Fig. 2G and Supplementary Fig. S1C). Similar results were observed in vivo. Immunofluorescence staining of livers from exosome-educated mice for CD206 revealed that exosomes derived from GC cells, particularly cells with high hepatic metastatic potential (MKN45-HL), significantly promoted CD206 expression compared with PBS (Fig. 2H and Supplementary Fig. S1D). The above results showed that GC-derived exosomes were mainly taken up by intrahepatic macrophages and could promote their transformation into the M2-like phenotype.

### GC-derived exosomes promote angiogenesis via M2-like macrophages to facilitate liver metastasis

**Fig. 3:**
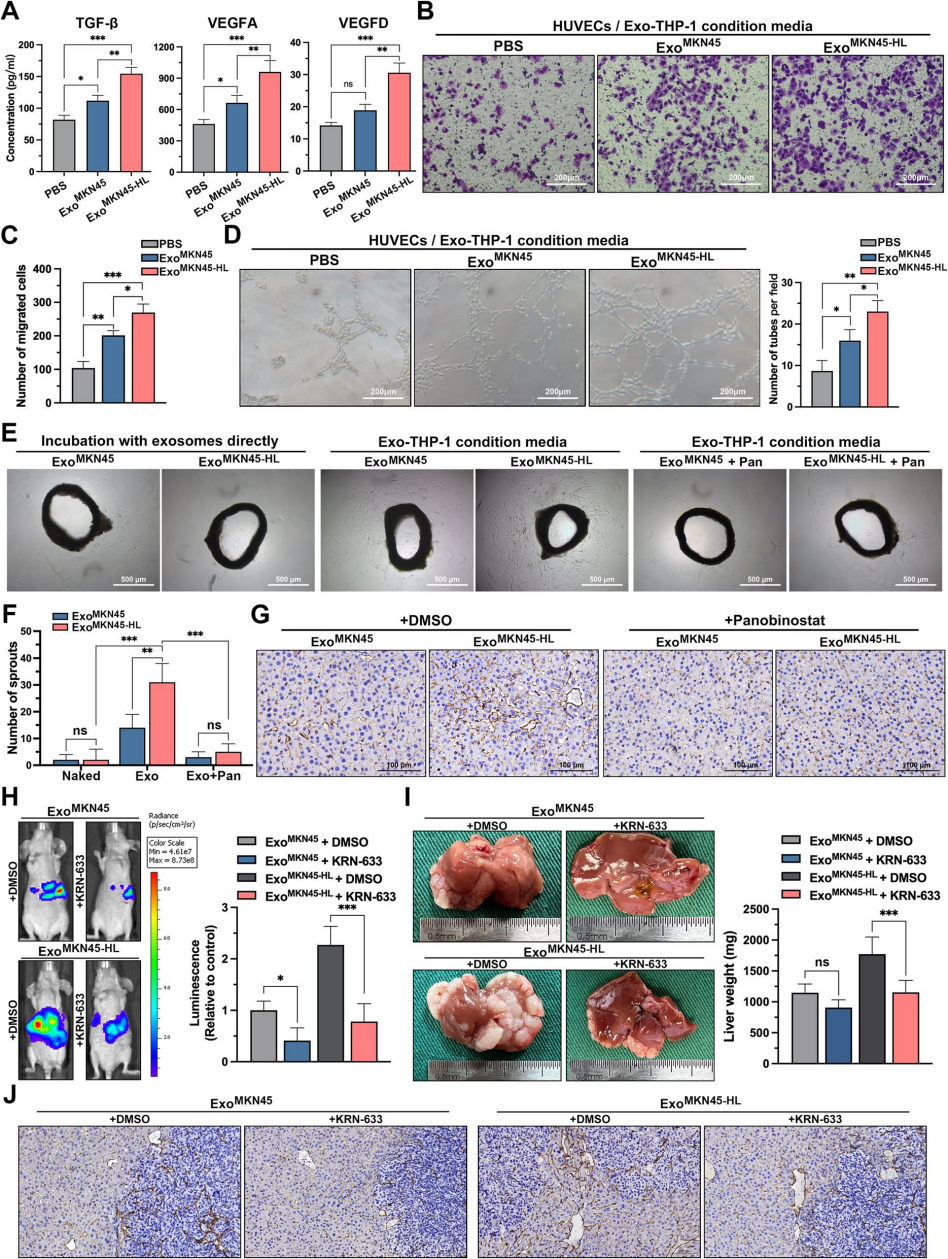
GC-derived exosomes promote angiogenesis via M2-like macrophages to facilitate liver metastasis. **A**. The supernatants from macrophages pre-incubated with GC cell-derived exosomes or PBS were examined to determine the secretion of TGF-β, VEGFA, and VEGFD by ELISA. **B**, **C**. Transwell assays evaluated the migration ability of HUVECs cocultured with macrophages treated with PBS, MKN45 exosomes or MKN45-HL exosomes. Representative images of migratory cells were shown. Scale bar = 200 μm. **D**. Tube formation of HUVECs cocultured with conditioned macrophages were determined. Scale bar = 200 μm. **E**, **F**. Aortic rings were cultured in condition media, and vascular outgrowth was quantified by counting all sprouts from one ring. Three culture environments were used: (Naked) exosomes were added directly to the culture medium of the vascular ring; (Exo) supernatant of PMA-treated THP-1 cells incubated with exosomes; (Exo + Pan) Panobinostat (0.0034 μM) was added while THP-1 was incubated with exosomes, and the supernatant was then extracted for culture of the vascular rings. Scale bar = 500 μm. **G**. Mice were administered Panobinostat (20 mg/kg every 2 days) intraperitoneally while receiving exosome education, and angiogenesis in the liver was observed two weeks later by immunohistochemistry with CD31. Scale bar = 100 μm. **H**. Mice educated with exosomes were simultaneously treated with KRN-633 (50 mg/kg, gastric lavage every 2 days) or DMSO followed by portal vein injection with luciferase-labeled MKN45 cells. The fluorescence signal in the metastases was detected and quantified using an in vivo imaging system (IVIS). **I**, **J**. The mice were then sacrificed, and their livers were photographed, weighed, and immunostained for CD31. Data are shown as mean ± standard deviation of 3 independent experiments, and statistical significance was determined using one-way ANOVA test (*P < 0.05, **P < 0.01, ***P < 0.001)

In metastatic target organs, angiogenesis forms the developmental basis for the transformation of dormant micrometastases into rapidly growing, clinically significant lesions, and M2-like macrophages have been reported to directly influence tumor cell survival, growth, and metastasis by promoting tumor angiogenesis [25]. In addition, TGF-β and VEGF have been shown to be involved in the activation, migration, tube formation, and maturation of the intercellular junctions and surrounding basement membrane of endothelial cells, thus playing an important regulatory role in tumor angiogenesis [26–30]. As shown in Fig. 3A, MKN45-HL-exo significantly increased the secretion of vascular-associated cytokines in PMA-treated THP-1 cells compared with PBS and MKN45-exo. Subsequently, the indirect in vitro co-culture systems were further applied to investigate the role of GC exosome-treated macrophages in angiogenesis (Supplementary Fig. S1E, F). Transwell assays indicated that the supernatants from PMA-treated THP-1 cells pre-incubated with MKN45-HL-exo significantly increased the migration of HUVECs (Fig. 3B, C). Tube formation assays and aortic ring assays were then performed to detect whether macrophages polarized by GC-derived exosomes affect angiogenesis. Compared with PBS and MKN45-exo, conditioned medium from PMA-treated THP-1 cells pre-incubated with MKN45-HL-exo significantly increased the number of tubules formed in HUVECs and sprouts from mouse aortic rings (Fig. 3D and Supplementary Fig. S1G, H). In addition, it was found that direct incubation of aortic rings with GC-derived exosomes or addition of Panobinostat [31] (an HDAC inhibitor that has been shown to strongly block M2-type polarization of macrophages in vivo and in vitro) to PMA-treated THP-1 cells did not promote sprouting of aortic rings (Fig. 3E, F). Subsequently, mice educated with exosomes were simultaneously administered Panobinostat or DMSO intraperitoneally. CD31 staining of the livers of these mice showed that MKN45-HL-derived exosomes significantly promoted the formation of disorganized neovascularizations in the livers of mice, but this pro-angiogenic effect was significantly diminished when the mice were treated with Panobinostat (Fig. 3G). This suggested that M2-like polarization of macrophages mediated by GC cell-derived exosomes is involved in the regulation of angiogenesis. To further investigate the effect of angiogenesis mediated by M2-like macrophages on GC-LM, KRN-633, a potent VEGF receptor inhibitor, was used in our exosome-educated GC-LM model. As shown in Fig. 3H, in vivo imaging of mice revealed that KRN-633 treatment significantly reduced the fluorescence intensity of mouse livers. Subsequently, the weight and H&E staining of the livers of the sacrificed mice exhibited consistent results (Fig. 3 I and Supplementary Fig. S1I). In addition, immunohistochemical staining for CD31 showed that treatment with KRN-633 markedly inhibited angiogenesis in and around liver metastases in mice (Fig. 3J). Collectively, these results indicated that GC-derived exosomes promote angiogenesis via M2-like macrophages to facilitate GC-LM.

### miR-519a-3p is highly expressed in exosomes from GC cells with high hepatic metastatic potential and can be transferred to macrophages via exosomes

**Fig. 4:**
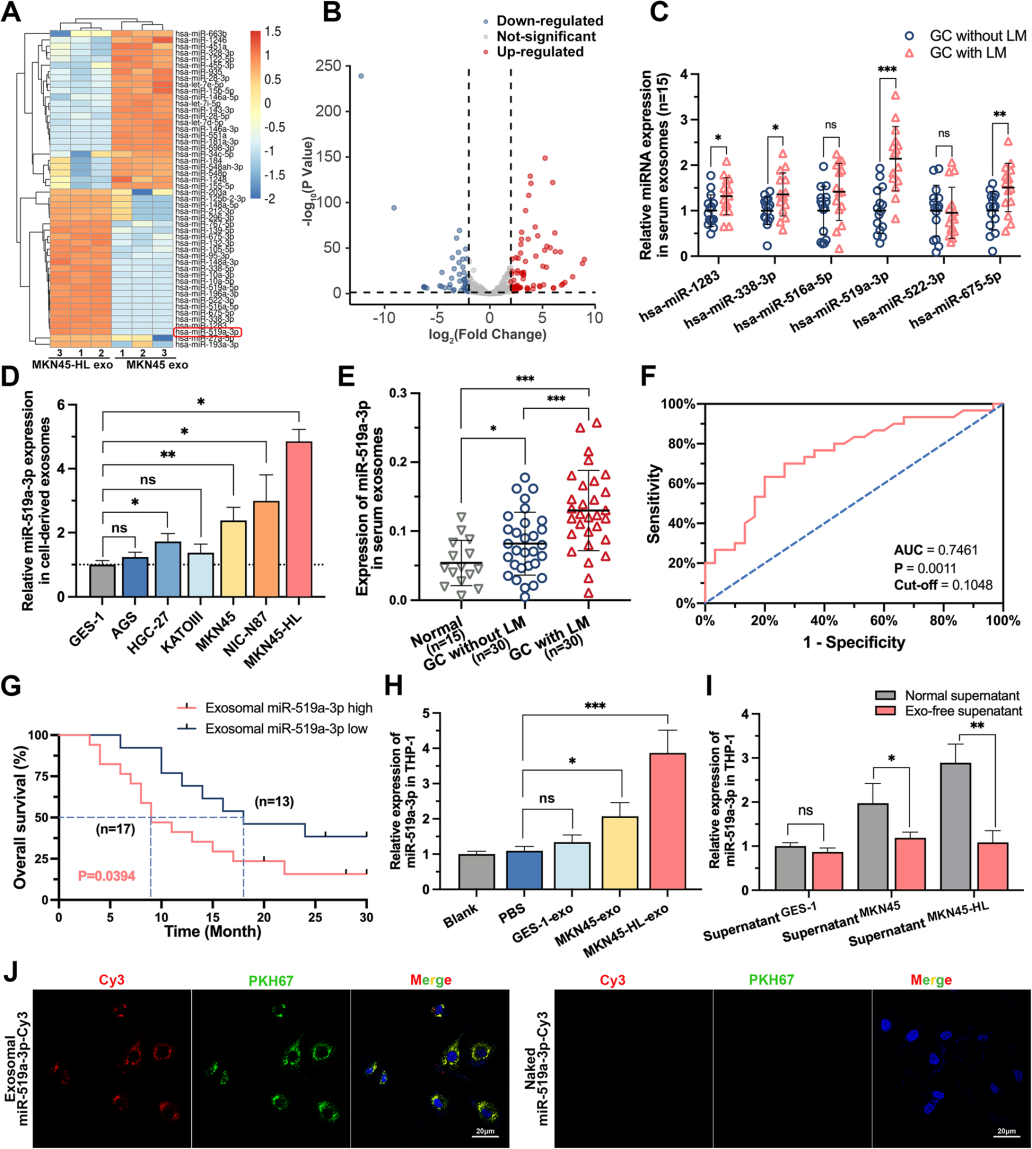
miR-519a-3p is highly expressed in exosomes from GC cells with high hepatic metastatic potential and can be transferred to macrophages via exosomes. **A**. Heatmaps of exosomal miRNAs show significant differences in the supernatant of MKN45-HL cells as compared to that of MKN45 cells (*n* = 3). **B**. Volcano plot of exo-miRNA-seq showed miRNAs with significant up- and down-regulation of expression. **C**. The top 6 upregulated miRNAs in exo-miRNA-seq were further validated by qRT-PCR in serum exosomes from GC patients with or without LM. **D**. The expression levels of miR-519a-3p in exosomes derived from six different GC cell lines and one normal gastric mucosal epithelial cell line were measured by qRT-PCR. **E**. Expression of exo-miR-519a-3p was detected by qRT-PCR in 30 GC patients with/without LM and 15 healthy volunteers. **F**. ROC Curve was generated based on exo-miR-519a-3p expression in serum from 30 GC-LM patients. **G**. KM plot was generated based on exo-miR-519a-3p expression in serum from 30 GC-LM patients. The cut-off value was determined based on the results of the ROC curve. **H**. PMA-treated THP-1 cells were incubated with PBS or GC cells (MKN45 and MKN45-HL)-derived exosomes for 48 h, and then miR-519a-3p expression was detected in THP-1 cells by qRT-PCR. **I**. PMA-treated THP-1 cells were cultured for 48 h with normal supernatants of GES -1, MKN45, and MKN45-HL cells and exosome-free supernatants (removed by ultracentrifugation). Subsequently, the expression of miR-519a-3p in THP-1 cells was detected by qRT-PCR. **J**. The left panels show the presence of Cy3 fluorescence and PKH67 lipid dye in PMA-treated THP-1 cells after addition of PKH67-labelled exosomes derived from MKN45 cells for 12 h. PMA-treated THP-1 cells incubated with naked-miR-519a-3p-Cy3 served as negative controls (the right panels). Scale bar = 20 μm. Data are shown as mean ± standard deviation of 3 independent experiments, and statistical significance was determined using Student’s t test, one-way ANOVA test, and log-rank analysis (*P < 0.05, **P < 0.01, ***P < 0.001)

The contents of exosomes, especially miRNA, play an important role in intercellular communication [32]. Therefore, exosomes isolated from MKN45 and MKN45-HL cells were subjected to miRNA sequencing to determine the targets involved in GC-LM (Fig. 4A). A total of 176 differentially expressed miRNAs were identified (Foldchange > 2 or < 0.5; FDR *p*-value < 0.05), including 71 down-regulated and 105 up-regulated miRNAs in MKN45-HL exosomes (Fig. 4B). The top 6 up-regulated miRNAs were selected for preliminary validation in serum exosomes from 15 GC-LM patients and 15 GC patients without LM, indicating that miR-519a-3p was the most upregulated miRNA (Fig. 4C and Supplementary Table S3). Subsequently, the expression of miR-519a-3p was detected, and the results showed that it was significantly upregulated in GC cells and tissues compared with normal gastric mucosal epithelial cells and tissues (Supplementary Fig. S2A-C). In addition, higher expression of miR-519a-3p was observed in GC-LM tissue compared with GC tissue without LM (Supplementary Fig. S2D-F). Subsequently, the expression of miR-519a-3p in exosomes (exo-miR-519a-3p) was further validated in GC cells and serum from expanded samples. The results showed that it was significantly highly expressed in cells with high LM potential and in serum from GC-LM (Fig. 4D, E). Moreover, the receiver operating characteristic curve (ROC) indicated that exo-miR-519a-3p expression in serum could serve as a potential diagnostic marker for GC-LM (Fig. 4F, AUC = 0.7461, *p* = 0.0011). Kaplan–Meier survival analysis showed that exo-miR-519a-3p expression in serum was negatively correlated with the endpoint of GC-LM patients (Fig. 4G, cut-off = 0.1048, *p* = 0.0426). An important function of exosomes is to influence cellular functions by delivering bioactive molecules to other cells [33]. In our previous study [21], GC-derived exosomes were found to be predominantly taken up by intrahepatic macrophages. Therefore, we next investigated whether miR-519a-3p in exosomes could also be delivered into macrophages via exosomes. As shown in Fig. 4H, PMA-treated THP-1 cells incubated with exosomes from MKN45-HL and MKN45 cells expressed more miR-519a-3p than with PBS. However, when the exosomes in the supernatant were removed by ultracentrifugation, the concentration of miR-519a-3p in PMA-treated THP-1 cells was not further increased (Fig. 4I). Moreover, it was observed by immunofluorescence that exo-miR-519a-3p labeled with Cy3 was internalized by PMA-treated THP-1 cells after coculture with them (Fig. 4J). These results indicate that miR-519a-3p is highly expressed in GC-derived exosomes, and its expression level in serum is closely associated with the prognosis of GC-LM patients and can be delivered to macrophages via exosomes.

### Exo-miR-519a-3p induces M2-like polarization of macrophages to promote angiogenesis and facilitate liver metastasis

**Fig. 5:**
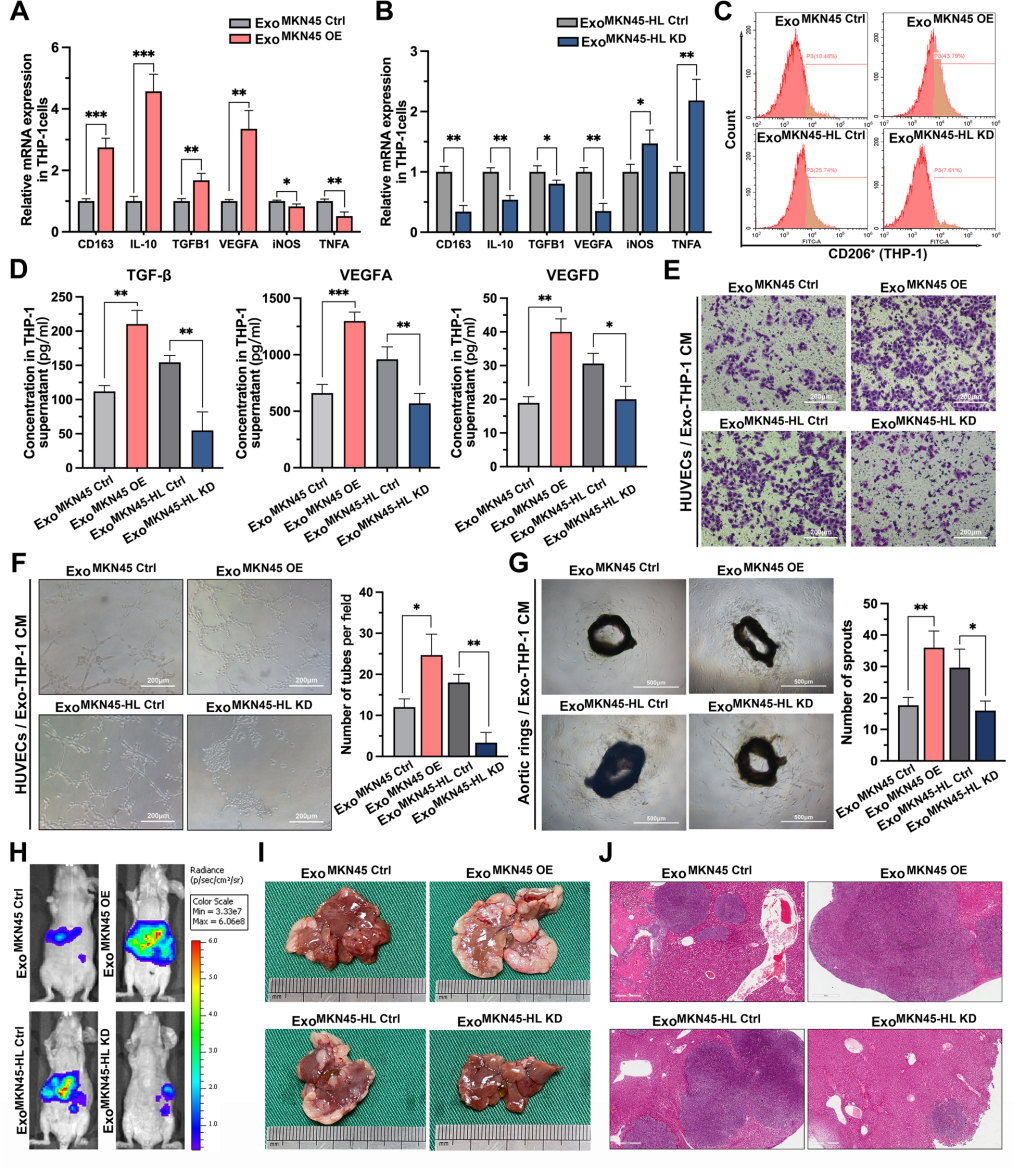
Exo-miR-519a-3p induces M2-like polarization of macrophages to promote agiogenesis and facilitate liver metastasis. **A**, **B**. PMA-treated THP-1 cells were incubated with exosomes from miR-519a-3p overexpressing MKN45 cells or miR-519a-3p knockdown MKN45-HL cells. The expression levels of CD163, IL-10, TGFB1, VEGFA, iNOS, and TNFA were subsequently measured by qRT-PCR. **C**. PMA-treated THP-1 cells were incubated with GC cell-derived exosomes with knockdown or overexpression of miR-519a-3p. The percentage of CD206-positive THP-1 cells were then measured by flow cytometry. **D**. The effect of exo-miR-519a-3p on the secretion of TGF-β, VEGFA, and VEGFD in PMA-treated THP-1 cells was determined by ELISA. **E**. Transwell assays of HUVECs were performed after 48 h of incubation with conditioned medium from PMA-treated THP-1 cells pre-incubated with miR-519a-3p-knockdown/overexpressed exosomes. Scale bar = 200 μm. **F**. Tubule formation assays of HUVECs were performed after incubation with conditioned medium from PMA-treated THP-1 cells. Scale bar = 200 μm. **G**. Effect of conditioned medium from PMA-treated THP-1 cells on vascular outgrowth of mouse aortic rings. Scale bar = 500 μm. **H**. Mice were educated with miR-519a-3p-inhibited/expressed exosomes from GC cells and then injected with luciferase-labeled MKN45 cells into the spleen. Liver metastasis was assessed 3 weeks later with IVIS. **I**, **J**. Mice from each of the above groups were sacrificed after completion of IVIS, and livers were collected for photographs and H&E staining. Scale bar = 500 μm. Data are shown as mean ± standard deviation of 3 independent experiments, and statistical significance was determined using Student’s t test and one-way ANOVA test (*P < 0.05, **P < 0.01, ***P < 0.001)

To investigate the role of exo-miR-519a-3p in GC-LM, MKN45 and MKN45-HL cell lines with stable overexpression and knockdown of miR-519a-3p, respectively, were constructed (Supplementary Fig. S3A, B). We then investigated whether aberrant intracellular miR-519a-3p expression could affect the biological behavior of MKN45 and MKN45-HL cells by CCK-8 assay, transwell assay, and subcutaneous implantation of xenograft tumors. The results showed that knockdown or overexpression of miR-519a-3p in MKN45 and MKN45-HL cells had little effect on the proliferation, migration, invasion, and subcutaneous tumor proliferation of GC cells (Supplementary Fig. S3C-H). In our study, GC-derived exosomes were shown to promote LM by inducing M2-like polarization of intrahepatic macrophages, so here we investigated whether this effect is mediated by miR-519a-3p in exosomes. The qRT-PCR results of PMA-treated THP-1 cells incubated with exosomes derived from miR-519a-3p-overexpressed MKN45 cells showed that the expression of M2 markers was dramatically upregulated and M1 markers were downregulated (Fig. 5A). The opposite trend of M2 and M1 marker expression was observed when PMA-treated THP-1 cells were incubated with miR-519a-3p-inhibited exosomes (Fig. 5B). In addition, detection of CD206 in exosome-incubated THP-1 cells by flow cytometry and in exosome-educated mouse liver by immunofluorescence showed that miR-519a-3p-knockdown exosomes suppressed CD206 expression, whereas miR-519a-3p-overexpressed exosomes promoted the expression of CD206 (Fig. 5C and Supplementary Fig. S4A-C). Subsequently, TGF-β, VEGFA, and VEGFD were detected by ELISA in the supernatant of PMA-treated THP-1 cells incubated with conditioned exosomes. This showed that miR-519a-3p-overexpressing and miR-519a-3p-suppressing exosomes increased and suppressed their expression, respectively, compared with the negative control (Fig. 5D). To determine the effect of exo-miR-519a-3p on angiogenesis, HUVECs were first co-cultured with PMA-treated THP-1 cells pre-incubated with exosomes. Transwell assays showed that THP-1 cells pre-incubated with miR-519a-3p-overexpressed and miR-519a-3p-inhibited exosomes increased and decreased the migratory capacity of HUVECs, respectively (Fig. 5E and Supplementary Fig. S4D). Consistent with the above observations, the conditioned medium of PMA-treated THP-1 cells pre-incubated with miR-519a-3p-overexpressed exosomes significantly increased the number of tubules formed by HUVECs and sprouts from aortic rings (Fig. 5F, G). Meanwhile, an exosome-educated GC-LM model was used to investigate the role of exo-miR-519a-3p in GC-LM. The results of IVIS showed that education with miR-519a-3p-overexpressed exosomes significantly increased the fluorescence signal in the liver compared with controls, whereas miR-519a-3p-suppressed exosomes attenuated the fluorescence signal (Fig. 5H and Supplementary Fig. S4E). Consistently, the weight and H&E staining of the livers of the sacrificed mice showed that education with miR-519a-3p-overexpressed exosomes promoted liver metastasis in mice compared with control exosomes, whereas LM was inhibited after education with miR-519a-3p-inhibited exosomes (Fig. 5I, J and Supplementary Fig. S4F). Furthermore, by immunohistochemical staining for CD31, we found that miR-519a-3p-overexpressing exosomes significantly promoted angiogenesis in liver metastases and surrounding tissues, whereas miR-519a-3p-suppressing exosomes inhibited angiogenesis compared with negative controls (Supplementary Fig. S4G). These results indicate that exo-miR-519a-3p induces polarization of M2 macrophages to promote angiogenesis and liver metastasis, whereas intracellular miR-519a-3p has a limited effect on the phenotype of primary GC cells.

### DUSP2-MAPK/ERK axis is the functional target of exo-miR-519a-3p in macrophages

**Fig. 6:**
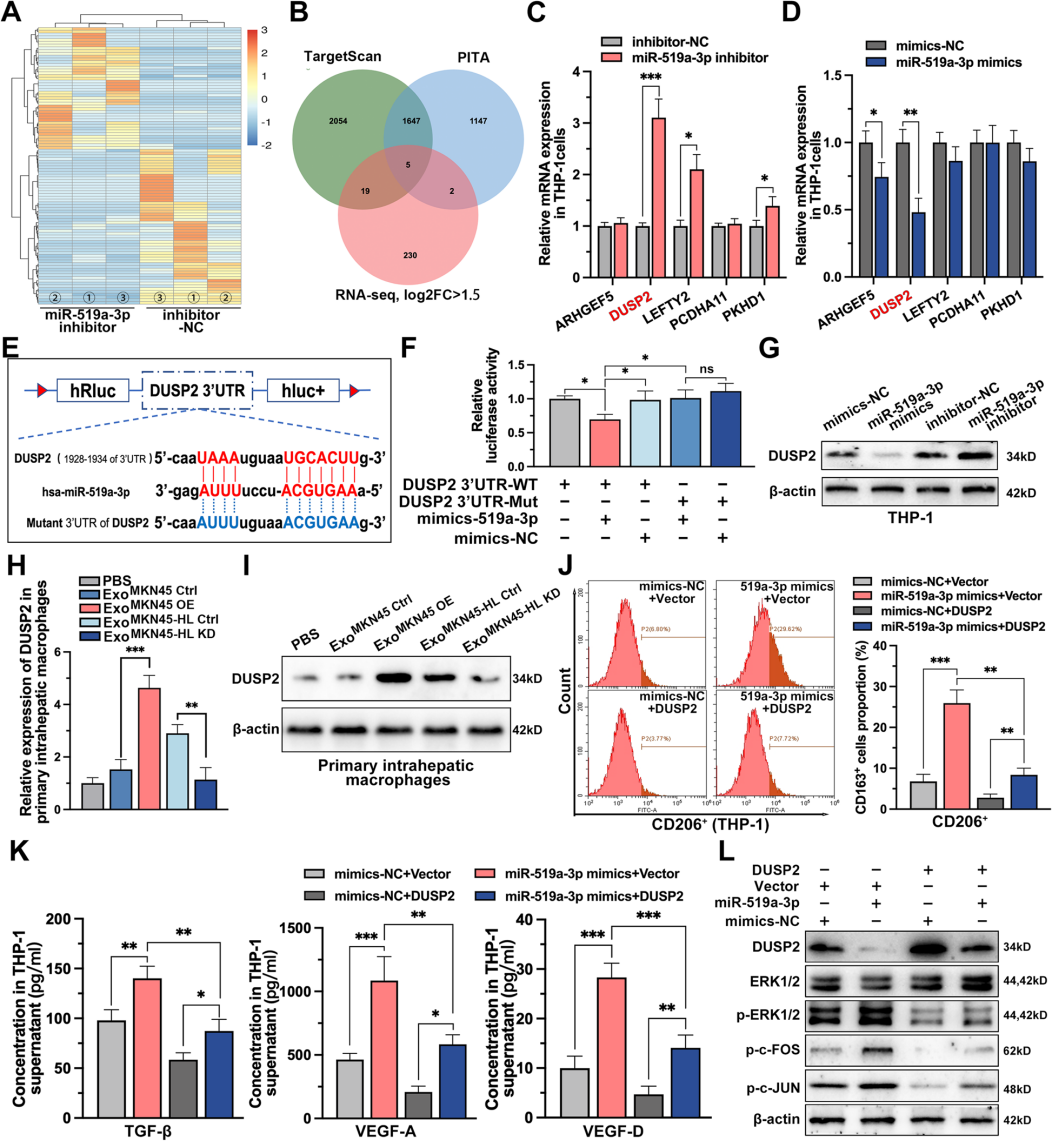
DUSP2-MAPK/ERK axis is the functional target of exo-miR-519a-3p in macrophages. **A**. mRNA-seq was performed in PMA-treated THP-1 cells with or without miR-519a-3p inhibitor. The heatmap showed the top 100 genes with significant differences in expression after knockdown of miR-519a-3p. **B**. Five mRNAs were identified that met the criteria to be upregulated (log_2_FC ≥ 1.5) based on mRNA-seq data and predicted to be miR-519a-3p targets based on TargetScan and PITA. **C**, **D**. The relative expression of the five targets in PMA-treated THP-1 cells transfected with miR-519a-3p NC/mimics or miR-519a-3p NC/inhibitors was detected by qRT-PCR. **E**. Schematic representation of the wild-type and mutant-type binding site between the 3'UTR of DUSP2 and miR-519a-3p. **F**. Relative luciferase activity of 3'UTR-DUSP2-luc constructs in HEK293T cells after transfection of miR-519a-3p mimics/NC. **G**. Expression of DUSP2 in PMA-treated THP-1 cells in which miR-519a-3p was overexpressed or knocked down was detected by Western blot. **H**, **I**. Expression of DUSP2 in primary liver macrophages from miR-519a-3p-deficient or enriched exosome-educated mice was detected by qRT-PCR and Western blot. **J**. Expression of CD206 (M2 marker) in miR-519a-3p overexpressing or miR-519a-3p/DUSP2 co-expressing PMA-treated THP-1 cells was determined by flow cytometry. **K**. Secretion of TGF-β, VEGFA, and VEGFD in miR-519a-3p overexpressing or miR-519a-3p/DUSP2 co-expressing PMA-treated THP-1 cells was detected by ELISA. **L**. Changes in the expression levels of the downstream cytokines p-ERK1/2, p–c-FOS, and p–c-JUN of the MAPK pathway was detected by Western blot in PMA-treated THP-1 cells transfected with miR-519a-3p mimics and DUSP2 overexpression vectors alone or together. Data are shown as mean ± standard deviation of 3 independent experiments, and statistical significance was determined using Student’s t test and one-way ANOVA test (*P < 0.05, **P < 0.01, ***P < 0.001)

To determine the downstream targets of miR-519a-3p, PMA-treated THP-1 cells transfected with miR-519a-3p inhibitor or negative controls were subjected to mRNA sequencing (Fig. 6A and Supplementary Table S4). And 5 genes potentially targeted by miR-519a-3p were identified by combining the genes upregulated in mRNA-seq (|log_2_Foldchange|> 1.5) with those predicted by the TargetScan and PITA databases (Fig. 6B). These five genes were further validated by qRT-PCR in PMA-treated THP-1 cells, and the results showed that only the expression of DUSP2 was significantly altered after knockdown and overexpression of miR-519a-3p (Fig. 6C, D). Based on the potential binding site of miR-519a-3p in the 3'UTR of DUSP2 predicted by bioinformatic analysis (Fig. 6E), luciferase reporter assays were performed. Luciferase activity in HEK293T cells was significantly attenuated when co-transfected with Luc-DUSP2-3'UTR-WT and miR-519a-3p mimics, whereas little change was observed when co-transfected with Luc-DUSP2-3'UTR-MUT and miR-519a-3p mimics, suggesting that miR-519a-3p directly targets DUSP2 (Fig. 6F). Then the results of Western blot revealed that mimics and inhibitors of miR-519a-3p transfected into PMA-treated THP-1 cells promoted and inhibited the expression of DUSP2, respectively (Fig. 6G). Similarly, DUSP2 was observed to be upregulated or downregulated in intrahepatic macrophages when mice were educated with miR-519a-3p-deficient or enriched exosomes, respectively (Fig. 6H, I). Moreover, the contribution of CD206 expression by miR-519a-3p mimics was found to be diminished by flow cytometry after co-transfection with DUSP overexpression plasmid (Fig. 6J). And it was observed that overexpression of DUSP2 in PMA-treated THP-1 cells impaired the pro-secretory effects of miR-519a-3p mimics on TGF-β, VEGF-A, and VEGF-D (Fig. 6K). Thus, miR-519a-3p induces M2-like polarization of macrophages and increases their secretion of angiogenesis-related factors by targeting DUSP2. DUSP2 is a nuclear phosphatase that specifically inactivates ERK by direct dephosphorylation of phosphothreonine and phosphotyrosine residues [34]. To investigate whether miR-519a-3p is involved in the activation of the MAPK/ERK pathway, key pathway-related proteins, including p-ERK1/2, p–c-FOS, and p–c- JUN, were measured by Western blot. The results showed that overexpression of miR-519a-3p in THP-1 cells significantly activated the MAPK/ERK pathway, but this activation was attenuated by overexpression of DUSP2 (Fig. 6L).

**Fig. 7:**
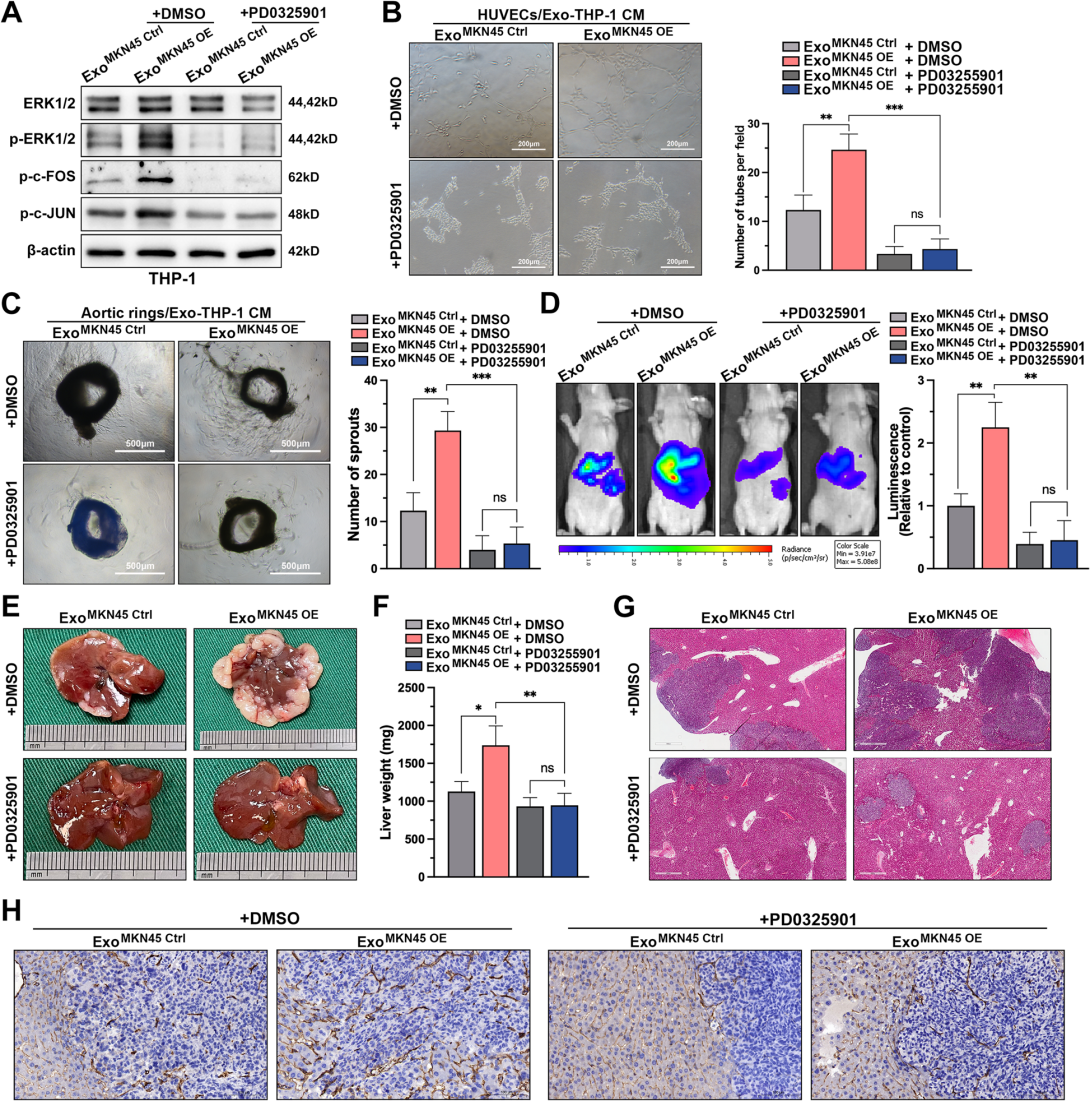
DUSP2-MAPK/ERK axis is the functional target of exo-miR-519a-3p in macrophages. **A**. Expression levels of downstream cytokines p-ERK1/2, p–c-FOS, and p–c-JUN in the MAPK pathway after addition of MEK1 inhibitor (PD0325901) to PMA-treated THP-1 cells pre-incubated with control or exosomes overexpressing miR-519a-3p. **B**. Tubule formation assays of HUVECs were performed after 48 h of incubation with conditioned medium (with or without MEK1 inhibitor) from PMA-treated THP-1 cells pre-incubated with exo-miR-519a-3p. Scale bar = 200 μm. **C**. Effect of conditioned medium (with or without PD0325901) of PMA-treated THP-1 cells pre-incubated with miR-519a-3p-riched or control exosomes on vascular sprouting in mouse aortic rings. Scale bar = 500 μm. **D**. Mice educated with miR-519a-3p-overexpressed or controlled exosomes were simultaneously treated with PD0325901 (20 mg/kg, gastric lavage every 2 days) or DMSO followed by portal vein injection with luciferase-labeled MKN45 cells. The fluorescence signal in the metastases was detected and quantified by IVIS. **E**–**H**. The mice were then sacrificed, and the livers were harvested for weighing, photography, H&E and CD31 staining. Scale bar = 500 μm. Data are shown as mean ± standard deviation of 3 independent experiments, and statistical significance was determined using one-way ANOVA test (*P < 0.05, **P < 0.01, ***P < 0.001)

The MAPK cascade is an important oncogenic factor in human cancers and is involved in M2-like activation of macrophages [35–38]. Therefore, targeting inhibition of this pathway is an important strategy for the treatment of GC-LM. PD0325901, a well-established non-ATP competitive MEK inhibitor with efficient inhibition of ERK1/2 phosphorylation, was used for our in vitro and in vivo experiments. The results of Western blot showed that PD0325901 treatment significantly inhibited the activation of MAPK/ERK pathway in THP-1 cells, and the addition of this inhibitor significantly blocked the activation of this pathway by exosome miR-519a-3p (Fig. 7A). Subsequently, the supernatants of PMA-treated THP-1 cells incubated with exosomes and treated with PD0325901 were used for angiogenesis assays and aortic ring assays. Supernatants of THP-1 cells preincubated with miR-519a-3p-rich exosomes significantly increased the number of tubules formed in HUVECs and the budding of aortic rings. However, when PMA-treated THP-1 cells were treated with PD0325901, exo-miR-519a-3p was barely able to exert a pro-angiogenic effect (Fig. 7B, C). Similar in vivo results were observed in the GC-LM model. We attempted to educate mice with miR-519a-3p-riched exosomes in the presence of inhibitors to partially promote LM of GC cells. However, the results of IVSI, H&E and CD31 staining of mouse liver showed that exo-miR-519a-3p could hardly exert its role in promoting angiogenesis and LM after oral administration of PD0325901 (Fig. 7D-H). Collectively, these results suggested that exo-miR-519a-3p creates a vascular-rich environment for the premetastatic niche by activating the DUSP2-MAPK/ERK axis in intrahepatic macrophages.

## Discussion

The liver is one of the most common organs of metastasis in GC patients. Approximately 4% to 14% of GC patients are diagnosed with LM at the time of initial diagnosis [2]. However, a comprehensive understanding of the underlying mechanisms of GC-LM is not yet available. In the present study, our results demonstrated that GC-derived miR-519a-3p-rich exosomes can be phagocytosed by intrahepatic macrophages and selectively initiate and establish a favorable pre-metastatic niche in the liver, thus playing a pivotal role in GC-LM.

Exosomes are extracellular vesicles rich in functional molecules such as proteins, lipids and nucleic acids [33]. In recent years, exosomes derived from tumors have been found to play a critical role in cell proliferation, metastasis, angiogenesis, drug resistance and immune escape [6, 39–41]. The hypoxic, chronic inflammatory microenvironment of the tumor was found to promote the release of exosomes [42]. The miRNAs and proteins in the exosomes are then taken up by target cells at a distant site and induced to transform into a pro-metastatic and pro-inflammatory phenotype by reprogramming or educating the target cells, which in turn forms a pre-metastatic niche. During cancer metastasis, exosomes have also been described as functional mediators with organ-specific properties in tumor-stroma interactions [43, 44]. In our study, GC-derived exosomes were mainly enriched in the liver, and further fluorescein tracing revealed that most exosomes were taken up by intrahepatic macrophages. Subsequent studies revealed that both PMA-treated THP-1 cells co-cultured with GC-derived exosomes and intrahepatic macrophages from exosome-educated mice exhibited phenotypic changes that favored tumor growth and metastasis. Although exosomes from MKN45-HL cells (with high liver metastatic capacity) did not show significant differences in size and number compared to exosomes from MKN45 cells, we did observe that the former exhibited a stronger pro-cancer capacity. Since the cargos in exosomes are the actual executors of their biological function and miRNAs are the major components [45], exosomal miRNA sequencing was performed. And we found that miR-519a-3p was significantly enriched in exosomes from MKN45-HL cells and in serum from GC-LM patients. This miRNA has already been shown to promote malignant biological behavior in various tumors [46–49], but the function of GC cell-derived exo-miR-519a-3p remains unclear, especially in GC-LM. Here, we found that miR-519a-3p in GC cells can be delivered to intrahepatic macrophages via exosomes to induce their polarization towards M2, facilitating the formation of pre-metastatic niches.

Cancer metastasis is a temporal and spatial process that begins with the formation of a pre-metastatic niche (a local environment suitable for the implantation and growth of circulating tumor cells) in distant tissues [17]. Macrophages play an important role in the formation of pre-metastatic niches [50]. J. E. Bader et al. found that depletion of macrophages in the late stage of tumorigenesis effectively reduced colon cancer growth in mice [51]. Similarly, N. Linde et al. observed that macrophages depletion inhibited the early dissemination of breast cancer and led to a reduced burden of lung metastases at the end stage of cancer progression [52]. However, the molecular mechanisms driving this pre-metastatic macrophage phenotype remain to be elucidated. Macrophages are defined as immunogenic M1 macrophages and "alternatively activated" or "repair" M2 macrophages based on their phagocytic and cytokine-producing properties [53]. In our study, we focused on the effects of GC-derived exosomes as well as exo-miR-519a-3p on phenotypic changes in intrahepatic macrophages. Using qRT-PCR, flow cytometry, and immunofluorescence to detect markers of macrophage polarization, we found that GC-derived exosomes, particularly miR-519a-3p in these exosomes, strongly induced the conversion of intrahepatic macrophages to the M2 phenotype. M2-polarised macrophages were found to promote angiogenesis by expressing more or different angiogenic factors [54]. Consistently, our results show that macrophages polarized to the M2 phenotype by exosomal miR-519a-3p secrete more pro-angiogenic cytokines, such as TGF-β, VEGFA and VEGFD. Moreover, supernatants from M2-polarized macrophages promoted migration and tubule formation of HUVECs and sprouting of aortic rings. Increased vascular permeability and neoangiogenesis facilitate tumor cell extravasation and subsequent metastatic growth in secondary organs and are therefore crucial steps in establishing a pre-metastatic ecological niche [55]. Our results demonstrate that miR-519a-3p-enriched exosomes promote pathological neoangiogenesis in the liver and that treatment with an angiogenesis inhibitor significantly impairs the ability of GC cells to form liver metastatic foci, indicating that intrahepatic M2-like macrophages activated by miR-519a-3p-rich exosomes play an important role in GC-LM by inducing pathological neovascularization. However, the underlying molecular mechanisms that trigger M2 polarization of macrophages in the liver need further investigation.

DUSP2 is a member of the dual specificity protein phosphatase subfamily and inhibits MAPK pathway activation mainly through dephosphorylation of p38 and ERK [56]. Since DUSP2 is mainly expressed in immune cells, most studies have focused on its role in regulating immunity and inflammation [57, 58]. Therefore, little is known about the potential mechanisms of DUSP2 in tumor progression, especially metastasis. Here we have identified DUSP2 as a functional target of exo-miR-519a-3p in liver macrophages, and its deletion in macrophages promotes activation of the MAPK/ERK pathway, which in turn induces macrophages to undergo M2-like polarization and paves the way for metastatic niche formation. Similar to the MAPK/ERK pathway, phosphorylation of STAT3 has also been confirmed to be a key factor involved in M2-like polarization of macrophages [59–62]. And recent studies have shown that DUSP2 inhibits the transcriptional activity of STAT3 by dephosphorylation at Tyr705 and Ser727 [63]. However, whether DUSP2 also promotes M2 polarization via p-STAT3 in hepatic macrophages remains to be investigated in detail. In general, targeting DUSP2 to inhibit M2 polarization of macrophages and weaken the pre-metastatic niche in the liver may be a novel strategy for the treatment of GC-LM. However, further informative studies are needed.

## Conclusion

**Fig. 8:**
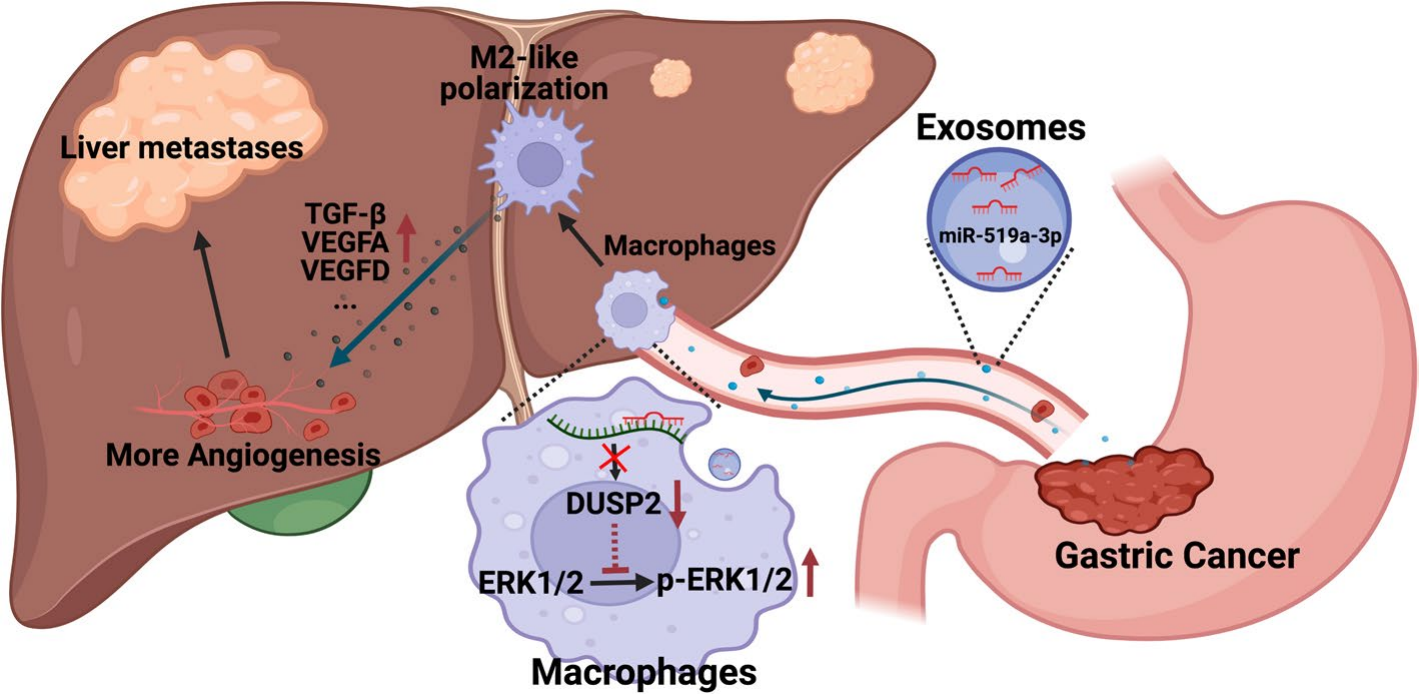
The mechanism diagram of GC-derived exo-miR-519a-3p on promoting liver metastasis via inducing intrahepatic M2-like macrophage-mediated angiogenesis

In summary, we propose a novel role for GC-derived exo-miR-519a-3p in the formation of angiogenesis-rich pre-metastatic niches by inducing M2 polarization in intrahepatic macrophages (Fig. 8). Our results also suggest that exo-miR-519a-3p in GC serum may be a novel biomarker for the diagnosis of GC-LM, and that treatment targeting exo-miR-519a-3p may prevent the formation of pre-metastatic niches in the liver and provide insights into anti-tumor metastasis therapy.

## Supplementary Information

**Additional file 1.**

**Additional file 2.**

**Additional file 3.**

## Data Availability

The exo-miRNA-seq data that support the findings of this study have been deposited in the SRA database from NCBI with the accession code PRJNA648286. Additional datasets used and/or analyzed during the current study are available from the corresponding author on reasonable request.

## Abbreviations

GC: Gastric cancer
LM: Liver metastasis
GC-LM: Gastric cancer with liver metastasis
miRNA: MicroRNA
KCs: Kupffer cells
Mo-Mfs: Monocyte-derived macrophages
mRNA-seq: MRNA sequencing
exo-miR-seq: Small RNA sequencing analysis of exosomes
MKN45-HL: MKN45 cell with highly hepatic metastatic potential
IVIS: in vivo Imaging system
PMA: 12-Myristate 13-acetate
TEM: Transmission electron microscopy
DUSP2: Dual specificity protein phosphatase 2
HUVECs: Human umbilical-vein endothelial cells
FISH: Fluorescence in situ hybridization
