# Supplementary material for: Gastric cancer-derived exosomal miR-519a-3p promotes liver metastasis by inducing intrahepatic M2-like macrophage-mediated angiogenesis
Source: J Exp Clin Cancer Res. 2022 Oct 10;41:296. doi: 10.1186/s13046-022-02499-8 (PMC9549645; doi:10.1186/s13046-022-02499-8)
Supplement: Supplementary file 1 — Additional file 1. [file 13046_2022_2499_MOESM1_ESM.pdf]

Supplementary Figure S1

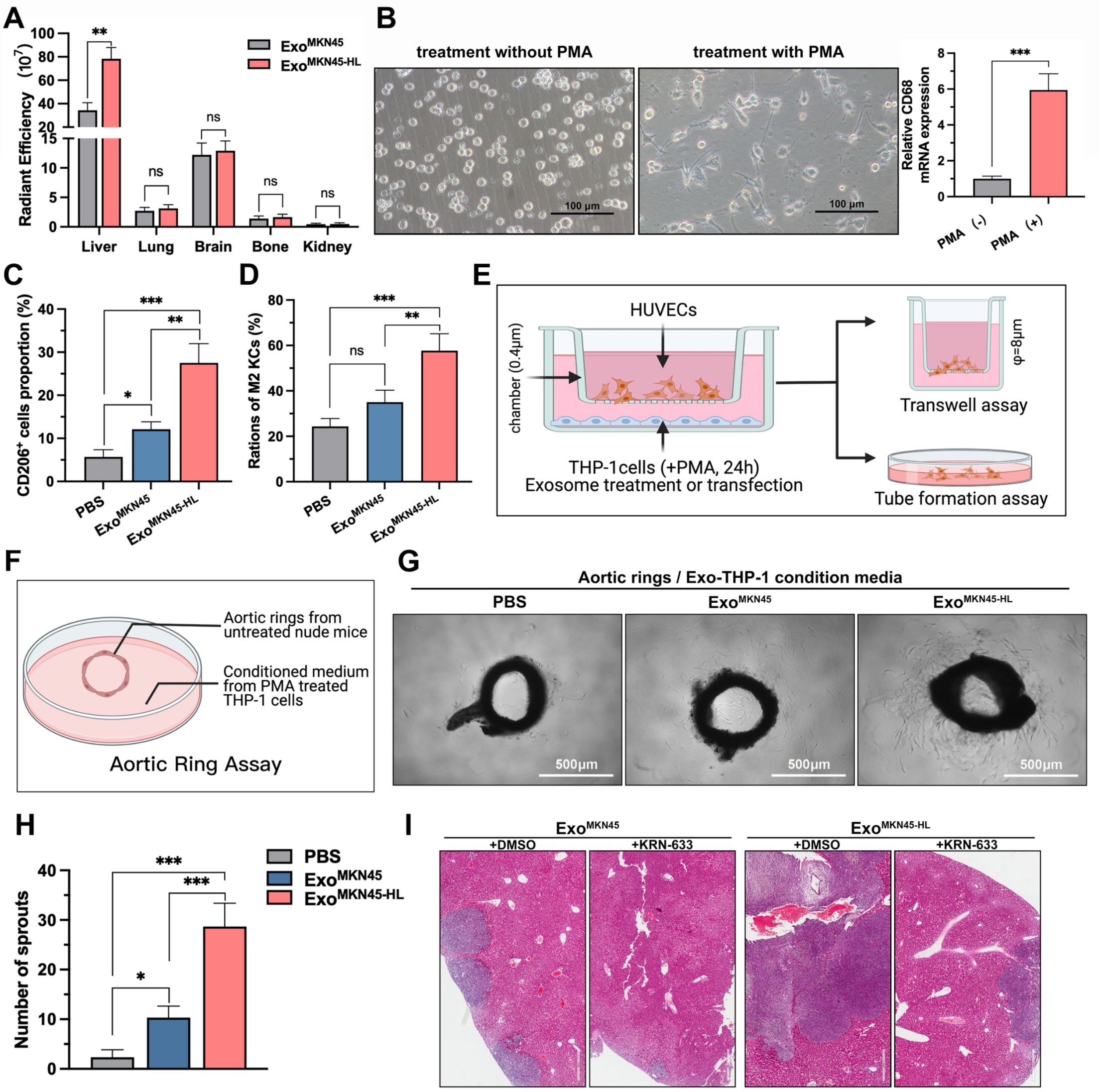

A. Statistical representation of the intensity of the fluorescence signal in the liver of mice educated with exosomes from MKN45 and MKN45-HL cells. **B.** Changes in morphology and CD68 expression (macrophage marker) of THP-1 cells after stimulation with 12-myristate (PMA) were examined by microscopy and qRT-PCR, respectively. **C.** The effect of GC cell-derived exosomes on the expression of CD206 (M2 marker) was detected by flow cytometry. **D.** Proportion of M2-like macrophages (F4/80$^+$ and CD206$^+$) in the liver of MKN45- and MKN45-HL- derived exosome-educated mice detected by immunofluorescence. **E.** Schematic diagram of the co-culture system and subsequent transwell assay and tube formation assay. The schematic was drawn using BioRender.com. **F.** Schematic diagram of the aortic ring assay. The schematic was drawn using BioRender.com. **G, H.** PMA-treated THP-1 cells were precultured with MKN45/MKN45-HL exosomes or PBS, and then

aortic rings were cultured with the supernatant of the cells. Vascular outgrowth was quantified by counting all sprouts from one ring. Scale bar=500 μm. **I.** Effect of GC-derived exosome education on liver metastasis in mice; representative photographs of H&E staining are shown, Scalebar = 500 μm. Data are shown as mean ± SD of 3 independent experiments, and statistical significance was determined using Student's t test and one-way ANOVA test (*P < 0.05, **P < 0.01, ***P < 0.001).

Supplementary Figure S2

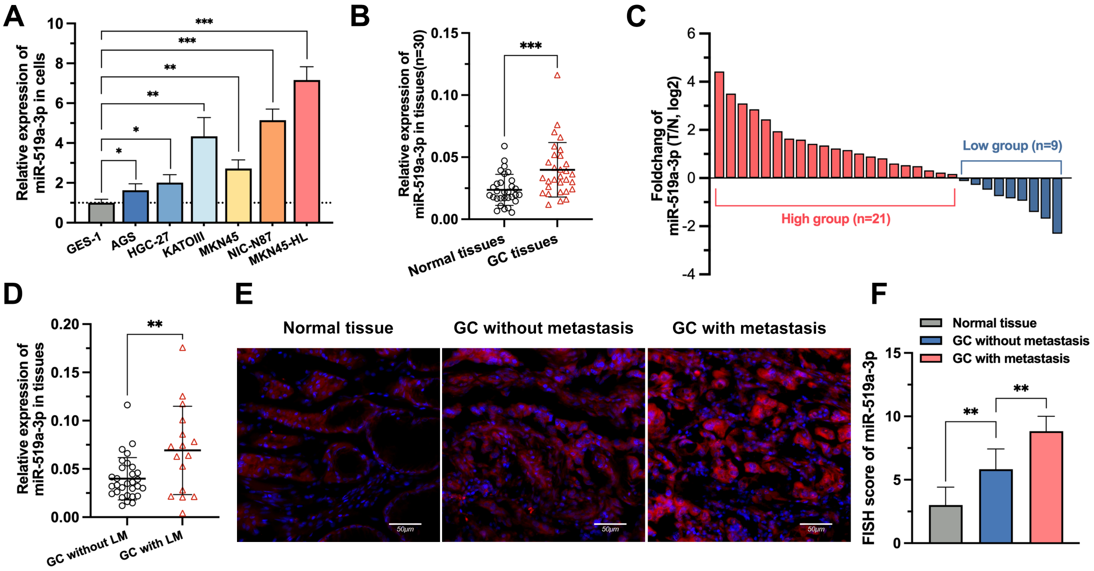

A. The qRT-PCR results showed that the expression of miR-519a-3p was significantly upregulated in GC cells compared with normal gastric mucosal epithelial cells (GES-1). **B.** Relative expression of miR-519a-3p in GC tissues and normal tissues. **C.** Fold change of miR-519a-3p in GC tissues compared with normal tissues. **D.** Relative expression of miR-519a-3p in GC tissues with/without LM. **E, F.** Higher expression of miR-519a-3p was detected by FISH in GC tissues with LM compared with GC tissues without LM and normal tissues (n=6), Scar bar = 50 μm. Data are shown as mean ± SD of 3 independent experiments, and statistical significance was determined using Student's t test and one-way ANOVA test (*P < 0.05, **P < 0.01, ***P < 0.001).

Supplementary Figure S3

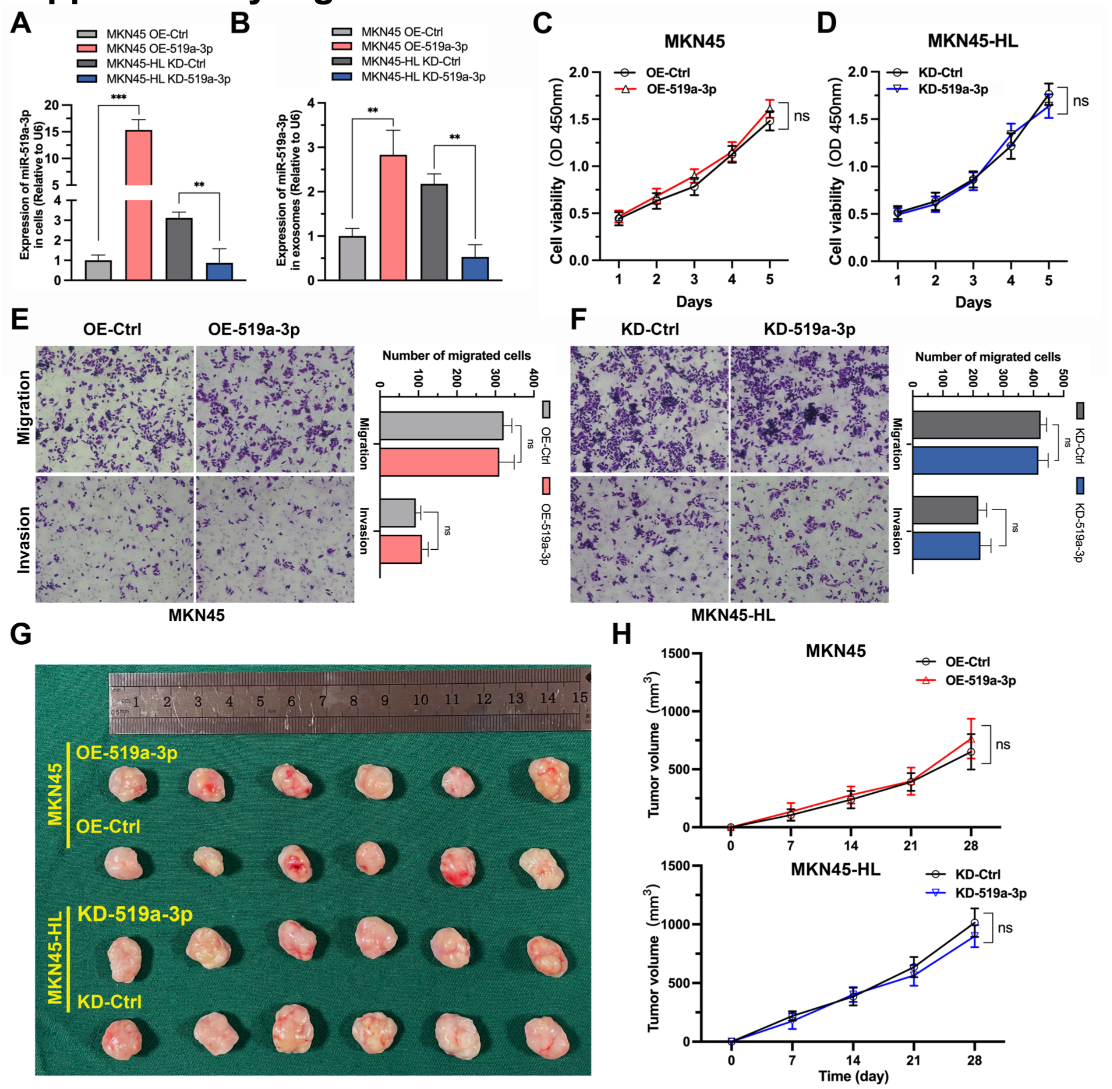

A,B. MKN45 and MKN45-HL cells with stable overexpression or knockdown of miR-519a-3p were constructed, and their efficiency in cells and exosomes was examined by qRT-PCR. **C.** Overexpression of miR-519a-3p showed little effect on MKN45 proliferation by CCK-8 assay. **D.** Suppression of miR-519a-3p had little effect on the proliferation of MKN45-HL cells by CCK-8 assay. **E.** Overexpression of miR-519a-3p in MKN45 cells hardly affects their migration and invasion. **F.** Knockdown of miR-519a-3p in MKN45-HL cells show little effect on their migration and invasion. **G,H.** Subcutaneous implantation of mice with GC cells stably knockdown or overexpressing miR-519a-3p, and proliferation of subcutaneous tumors proved to be almost no difference. Data are shown as mean ± SD of 3 independent experiments, and statistical significance was determined using Student's t test and one-way ANOVA test (*P < 0.05, **P < 0.01, ***P < 0.001).

Supplementary Figure S4

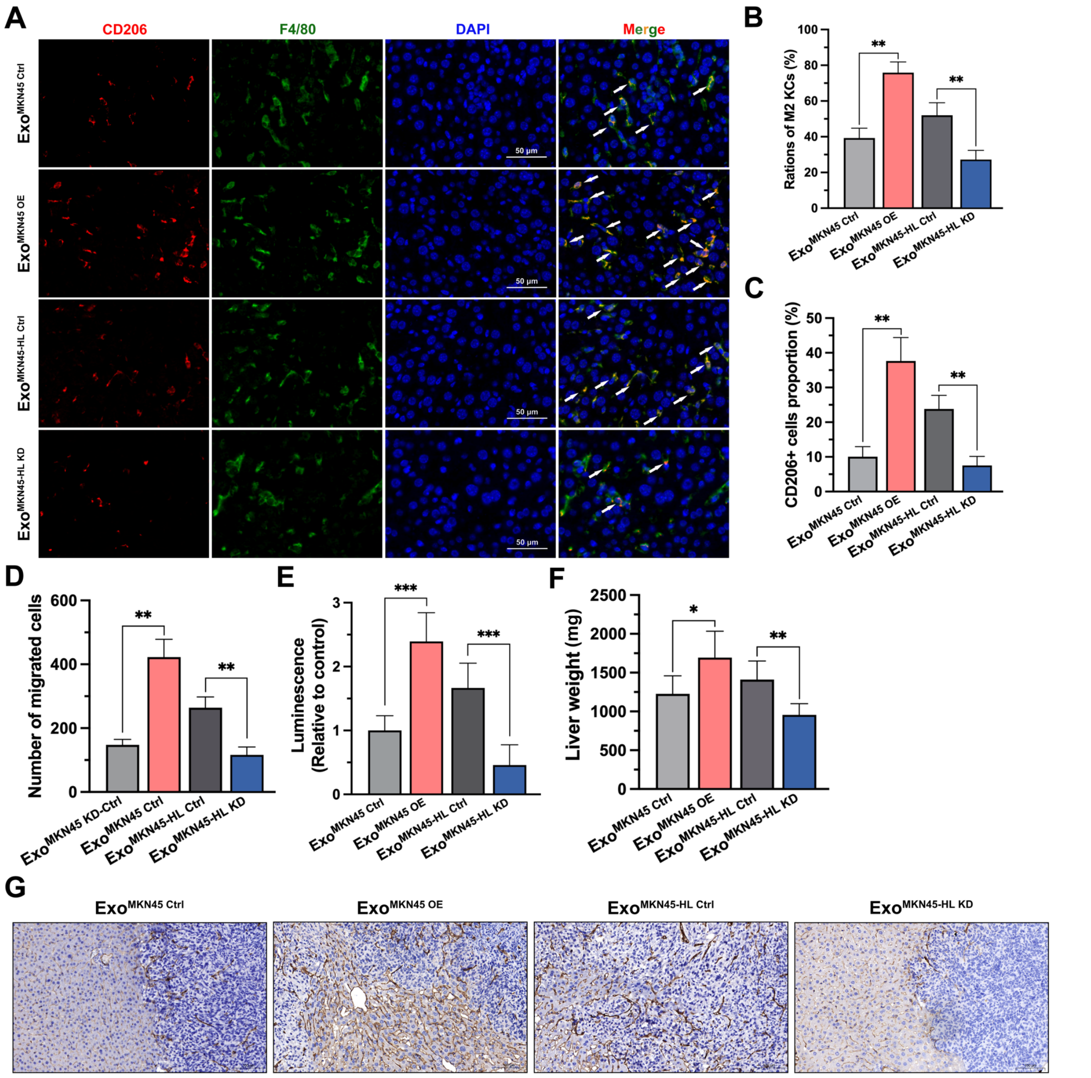

A, B. Mice were educated with miR-519a-3p-overexpressed/inhibited exosomes, then the expression of CD206 in liver macrophages (F4/80) were detected by immunofluorescence. Scale bar=50 μm. **B.** The percentage of CD206 (+) THP-1 cells induced with different exosomes was detected and analyzed by flow cytometry. **D.** The results of transwell assays were statistically analyzed to detect the effect of exo-miR-519a-3p on HUVECs. **E,F.** Statistical analysis of fluorescence signal intensity and weight in mouse liver to evaluate the effect of exo-miR-519a-3p on LM. **G.** Representative images of CD31 immunohistochemical staining of LM tissues from mice educated with miR-519a-3p-inhibited/expressed exosomes, scale bar=100 μm. Data are shown as mean ± SD of 3 independent experiments, and statistical significance was determined using Student's t test (*P < 0.05, **P < 0.01, ***P < 0.001).

Supplementary Table S1

Antibodies used in the present study		
**Product**	**Source**	**No. of Catalogue**
**Western blot**		
Primary antibody		
Anti-CD81	Abcam	Ab79599
Anti-TSG101	Abcam	Ab125011
Anti-Calnexin	Abcam	Ab22595
Anti-β-actin	Proteintech	66009-1-Ig
Anti-DUSP2	Abcam	Ab137640
Anti-ERK1/2	Abcam	Ab184699
Anti-p-ERK1/2	Cell Signaling Technology	#9101
Anti-p-c-FOS	Cell Signaling Technology	#5348
Anti-p-c-JUN	Abcam	ab32385
Secondary antibody		
Anti-rabbit IgG-HRP	Proteintech	SA00001-15
Anti-mouse IgG-HRP	Proteintech	SA00001-1
**IHC and IF**		
Primary antibody		
Anti-CD31	Cell Signaling Technology	#3528
Anti-F4/80	Santa Cruz	Sc-377009
Anti-CD206	Cell Signaling Technology	#24595
Secondary antibody		
Goat Anti-Mouse IgG H&L (Alexa Fluor® 647)	Abcam	Ab150115
Goat Anti-Rabbit IgG H&L (Alexa Fluor® 594)	Abcam	Ab150080

Supplementary Table S2

Primer, probes, and siRNAs used in the experiments

Gene name	Sequence (5'-3')
qRT-PCR primers:	
CD68	F: GGAAATGCCACGGTTCATCCA
	R: TGGGGTTCAGTACAGAGATGC
CD163	F: TTTGTCAACTTGAGTCCCTTCAC
	R: TCCCGCTACACTTGTTTTCAC
IL-10	F: GACTTTAAGGGTTACCTGGGTTG
	R: TCACATGCGCCTTGATGTCTG
TGFB1	F: GGCCAGATCCTGTCCAAGC
	R: GTGGGTTTCCACCATTAGCAC
VEGFA	F: AGGGCAGAATCATCACGAAGT
	R: AGGGTCTCGATTGGATGGCA
iNOS	F: TTCAGTATCACAACCTCAGCAAG
	R: TGGACCTGCAAGTTAAAATCCC
TNF	F: CCTCTCTCTAATCAGCCCTCTG
	R: GAGGACCTGGGAGTAGATGAG
DUSP2	F: GGGCTCCTGTCTACGACCA
	R: GCAGGTCTGACGAGTGACTG
β-actin	F: CCCCAGGCACCAGGGCGTGAT
	R: GTCATCTTCTCGCGGTTGGCCTTGGGGT
GAPDH	F: CGGAGTCAACGGATTTGGTCGTAT
	R: AGCCTTCTCCATGGTGGTGAAGAC
hsa-miR-1283	F: CGCGCTCTACAAAGGAAAGCG
	R: CAGTGCGTGTCGTGGAGT
hsa-miR-338-3p	F: CAGCCACCCACTCAGAGCG
	R: GTGGCTCTGAGAATCTTCG
hsa-miR-516a-5p	F: TCGGCAGGUUCUCGAGGAAA
	R: CACTCAACTGGTGTCGTGGA
hsa-miR-519a-3p	F: TGCGGGTGCTCGCTTCGGCAGC
	R: GTGCAGGGTCCGAGGTATT
hsa-miR-522-3p	F: CTCTAGAGGGAAGCGCTTTCTG
	R: GAAAGCGCTTCCCTCTAGAGTT
hsa-miR-675-5p	F: TGGTGCGGAGAGGGCCCACAGTG
	R: TGGTGTCGTGGAGTCG
U6	F: CTCGCTTCGGCAGCACA
	R: AACGCTTCACGAATTTGCGT
mimics and inhibitors:	
miR-519a-3p mimics	AAAGUGCAUCCUUUUAGAGUGU
mimic NC	UUCUCCGAACGUGUCACGUUU
miR-519a-3p inhibitors	ACACUCUAAAAGGAUGCACUUU
inhibitor NC	ACACUCUAAAAGGAACGUGAAU
probes:	
miR-519a-3p probes	ACACUCUAAAAGGAUGCACUUU-/cy3/
