# Supplementary material for: Gastric cancer-derived exosomal miR-519a-3p promotes liver metastasis by inducing intrahepatic M2-like macrophage-mediated angiogenesis
Source: J Exp Clin Cancer Res. 2022 Oct 10;41:296. doi: 10.1186/s13046-022-02499-8 (PMC9549645; doi:10.1186/s13046-022-02499-8)
Supplement: Supplementary file 2 — Additional file 2. [file 13046_2022_2499_MOESM2_ESM.pdf]

ID	logFC	logCPM	PValue	FDR
hsa-miR-516a-5p	8.97169363	6.95437622	3.21E-38	3.08E-37
hsa-miR-522-3p	8.78928566	6.77240147	8.71E-34	7.46E-33
hsa-miR-1283	7.85569	5.89758682	3.40E-19	1.71E-18
hsa-miR-675-5p	7.12970647	5.29163616	5.79E-13	2.21E-12
hsa-miR-338-3p	6.92564318	5.12816379	3.78E-11	1.35E-10
hsa-miR-519a-3p	6.80385208	5.01705696	4.82E-10	1.59E-09
hsa-miR-148a-5p	6.70946717	7.20218449	1.52E-46	2.09E-45
hsa-miR-10a-3p	6.21084894	7.20641755	7.50E-47	1.08E-45
hsa-miR-203a	6.16748887	8.45448104	2.34E-67	6.17E-66
hsa-miR-519a-5p	6.04140726	4.48655578	5.71E-07	1.45E-06
hsa-miR-196a-3p	6.03987887	4.48685987	7.80E-07	1.96E-06
hsa-miR-148a-3p	5.96656369	15.5450524	9.90E-123	7.85E-121
hsa-miR-139-5p	5.72933388	7.39289677	4.48E-51	7.47E-50
hsa-miR-338-5p	5.41321145	8.03073935	1.11E-52	1.95E-51
hsa-miR-27a-5p	5.36397297	6.4113825	9.20E-30	7.29E-29
hsa-miR-10a-5p	5.24473417	12.4468775	1.75E-149	2.78E-147
hsa-miR-105-5p	5.20769822	7.52007786	2.20E-48	3.32E-47
hsa-miR-212-3p	5.10332164	5.07575309	2.05E-10	6.90E-10
hsa-miR-675-3p	5.03023685	6.97394304	1.02E-30	8.52E-30
hsa-miR-125b-2-3p	4.75005348	4.83698736	2.17E-08	6.25E-08
hsa-miR-296-3p	4.72207593	4.80835131	3.08E-09	9.69E-09
hsa-miR-132-3p	4.54343821	9.07279725	3.70E-64	9.03E-63
hsa-miR-767-5p	4.36519267	7.49346022	7.01E-42	8.54E-41
hsa-miR-95-3p	4.30026438	9.60259247	6.87E-74	2.42E-72
hsa-miR-193a-3p	4.14418426	4.97684056	1.11E-09	3.60E-09
hsa-miR-212-5p	4.09430777	4.39743274	6.25E-06	1.47E-05
hsa-miR-27a-3p	3.90252782	14.8141236	9.11E-122	5.77E-120
hsa-miR-20a-5p	3.83363263	13.8805897	1.05E-129	1.11E-127
hsa-miR-17-5p	3.69200964	12.7084573	1.39E-108	7.34E-107
hsa-miR-24-2-5p	3.63966328	9.61502667	1.53E-60	3.24E-59
hsa-miR-188-5p	3.61229463	4.61832474	1.24E-05	2.83E-05
hsa-miR-106a-5p	3.54831179	4.57397724	9.21E-06	2.11E-05
hsa-miR-296-5p	3.54697725	4.93792239	2.62E-07	6.93E-07
hsa-miR-30a-5p	3.39086795	14.8228056	7.09E-92	2.81E-90
hsa-miR-582-3p	3.3765649	8.68016451	4.34E-36	4.05E-35
hsa-miR-148b-5p	3.20606329	7.18419042	3.49E-24	2.31E-23
hsa-miR-365a-3p	3.19723157	9.05111693	3.11E-21	1.67E-20
hsa-miR-365b-3p	3.1972281	9.05111693	3.27E-21	1.73E-20
hsa-miR-132-5p	3.1595274	6.41844964	5.46E-17	2.44E-16
hsa-miR-181c-5p	3.10200939	7.41856051	1.47E-23	8.94E-23
hsa-miR-24-3p	3.01398971	14.4652848	3.66E-68	1.05E-66
hsa-miR-1291	3.00639667	7.81924	6.82E-23	3.93E-22
hsa-miR-193b-3p	3.00084614	11.4842427	2.21E-60	4.38E-59
hsa-miR-23a-3p	2.94229734	14.1457247	7.45E-55	1.39E-53
hsa-miR-182-5p	2.91662365	11.4232812	2.83E-45	3.59E-44
hsa-miR-183-5p	2.68064308	10.7425148	1.38E-39	1.51E-38
hsa-miR-330-3p	2.63528633	8.03170388	6.81E-26	4.79E-25
hsa-miR-193b-5p	2.60416808	5.68848754	6.39E-10	2.09E-09
hsa-miR-10b-5p	2.58680605	9.01737006	2.84E-23	1.70E-22
hsa-miR-125b-1-3p	2.5350982	4.570913	0.0002798	0.0005543
hsa-miR-4449	2.52542894	6.00949711	3.24E-08	9.16E-08

hsa-miR-365a-5p	2.52398124	5.40041135	4.58E-05	0.0001015
hsa-miR-330-5p	2.5045915	6.1457228	4.47E-09	1.38E-08
hsa-miR-30a-3p	2.48561102	10.7108776	3.05E-40	3.59E-39
hsa-miR-181c-3p	2.26280938	6.97242074	2.35E-15	9.92E-15
hsa-miR-1250-5p	2.22836422	4.60986242	0.0029833	0.005254
hsa-miR-4326	2.2109864	6.01883369	1.77E-08	5.16E-08
hsa-miR-19b-3p	2.11155937	10.8396125	3.17E-25	2.14E-24
hsa-miR-148b-3p	2.10194569	11.647217	7.71E-39	7.89E-38
hsa-miR-584-5p	2.07683857	5.32719312	8.91E-06	2.06E-05
hsa-miR-660-5p	2.05972988	8.82255519	1.93E-22	1.07E-21
hsa-miR-193a-5p	2.03129237	6.14663	5.85E-08	1.63E-07
hsa-miR-18a-5p	2.01261317	7.08362459	9.79E-14	3.79E-13
hsa-miR-30d-5p	1.97126681	13.6533165	2.46E-29	1.90E-28
hsa-miR-99a-5p	1.9525308	10.5606258	2.50E-14	1.03E-13
hsa-miR-125b-5p	1.88279048	9.88239626	1.17E-25	8.10E-25
hsa-miR-3648	1.84973382	6.1350714	1.23E-06	3.00E-06
hsa-miR-196a-5p	1.84138833	12.0261738	3.55E-28	2.68E-27
hsa-miR-92a-3p	1.83782047	14.630167	1.37E-23	8.49E-23
hsa-miR-1277-5p	1.82266277	4.39541305	0.018666	0.0288641
hsa-miR-93-5p	1.77848148	13.4159255	3.42E-23	2.01E-22
hsa-miR-4454	1.71983776	5.61594604	5.68E-05	0.0001233
hsa-miR-191-5p	1.69333319	13.6520206	8.45E-19	4.18E-18
hsa-miR-152-3p	1.64461969	9.78655896	4.23E-14	1.70E-13
hsa-miR-30c-5p	1.63333861	11.6099778	1.60E-22	9.08E-22
hsa-miR-181d-5p	1.62857975	10.5086852	9.53E-21	4.95E-20
hsa-miR-4488	1.60308112	5.14542564	0.0016887	0.0030415
hsa-miR-629-5p	1.50921004	8.0117675	2.38E-11	8.56E-11
hsa-let-7b-3p	1.45757862	5.71634448	0.0001977	0.0004043
hsa-miR-100-5p	1.45546884	12.9377202	1.44E-18	7.00E-18
hsa-miR-760	1.44434454	4.64245397	0.0187861	0.0289086
hsa-miR-542-3p	1.40804029	6.04479074	0.0001786	0.0003677
hsa-miR-532-5p	1.38052352	11.020051	2.53E-18	1.22E-17
hsa-miR-452-3p	1.33422073	8.28006663	4.13E-09	1.28E-08
hsa-miR-30d-3p	1.32415439	5.32104351	0.0148557	0.0233131
hsa-miR-7704	1.30008496	7.9429167	2.44E-05	5.53E-05
hsa-miR-222-3p	1.280666	10.968395	6.12E-14	2.42E-13
hsa-miR-30b-5p	1.27175079	10.455988	4.11E-14	1.67E-13
hsa-miR-130a-3p	1.20254321	7.91545914	4.81E-07	1.24E-06
hsa-miR-425-3p	1.20162259	7.72246016	1.42E-06	3.43E-06
hsa-miR-331-5p	1.19907837	5.87328445	0.0007742	0.0014784
hsa-miR-17-3p	1.19053865	4.64387239	0.1131389	0.1552599
hsa-miR-140-3p	1.18835349	10.9340732	4.84E-09	1.48E-08
hsa-miR-18a-3p	1.1866023	5.43666814	0.0152948	0.0238839
hsa-miR-501-3p	1.18118337	6.07630941	0.0010059	0.0018647
hsa-miR-424-5p	1.17008909	7.82034275	7.91E-07	1.98E-06
hsa-miR-425-5p	1.15280475	10.0248608	3.59E-10	1.20E-09
hsa-let-7f-2-3p	1.11252899	4.60978948	0.1273347	0.1710385
hsa-miR-192-5p	1.10453713	11.0423791	4.94E-12	1.80E-11
hsa-miR-671-5p	1.10018555	5.39461116	0.0156674	0.0243459
hsa-miR-22-3p	1.0696644	10.9544872	9.09E-08	2.48E-07
hsa-miR-941	1.06494516	9.3079386	4.85E-07	1.24E-06
hsa-miR-29c-3p	1.06199647	5.62624653	0.0525134	0.0749854

hsa-miR-151a-3p	1.04261862	12.7989562	1.42E-07	3.84E-07
hsa-miR-502-3p	1.03440373	6.40562963	0.0076751	0.0127382
hsa-miR-450a-5p	-1.0010251	7.46414103	8.31E-05	0.0001749
hsa-miR-98-5p	-1.0078164	10.683338	6.06E-09	1.83E-08
hsa-miR-331-3p	-1.0166738	4.81076435	0.1185951	0.1613505
hsa-miR-877-5p	-1.0282469	5.25079889	0.0388291	0.0564625
hsa-miR-589-5p	-1.1167304	7.72349138	0.0002106	0.0004263
hsa-miR-25-5p	-1.2174413	4.46563625	0.1354731	0.1810477
hsa-miR-378a-5p	-1.2983607	5.41659769	0.0099357	0.0159879
hsa-miR-663a	-1.3168773	7.11750099	0.002522	0.0044663
hsa-miR-374b-3p	-1.334924	4.84749101	0.0399373	0.0575461
hsa-miR-149-5p	-1.3400841	8.94532753	6.23E-11	2.19E-10
hsa-miR-424-3p	-1.3506928	6.611821	8.72E-05	0.0001819
hsa-miR-335-3p	-1.3536538	6.1742719	0.0002423	0.0004861
hsa-miR-942-5p	-1.3629867	5.00311596	0.0092212	0.0150677
hsa-miR-744-5p	-1.3693663	8.8567423	2.53E-09	8.09E-09
hsa-miR-92b-5p	-1.4117622	5.96917787	0.000618	0.0011945
hsa-miR-499a-5p	-1.4134152	5.03560429	0.0147185	0.0232127
hsa-miR-27b-5p	-1.4695114	5.0564326	0.0230241	0.0349218
hsa-miR-454-3p	-1.4844247	8.32045915	6.64E-11	2.31E-10
hsa-miR-874-3p	-1.5333828	5.56008882	0.0010993	0.0020144
hsa-let-7b-5p	-1.5456009	14.1512716	2.32E-26	1.67E-25
hsa-miR-455-5p	-1.6424706	9.13354039	7.29E-14	2.85E-13
hsa-miR-1301-3p	-1.6551142	4.87927058	0.0058938	0.0101541
hsa-miR-185-3p	-1.6922124	4.74000515	0.0251035	0.0377147
hsa-miR-345-5p	-1.820634	7.65895958	2.90E-12	1.07E-11
hsa-miR-769-5p	-1.8487205	8.79353164	7.28E-18	3.40E-17
hsa-miR-486-5p	-1.8505806	7.15571945	1.28E-08	3.82E-08
hsa-miR-590-3p	-1.8628791	6.85830297	1.57E-08	4.60E-08
hsa-let-7f-5p	-1.9368123	14.8892719	7.05E-36	6.39E-35
hsa-miR-423-5p	-1.9690986	10.515723	8.11E-18	3.73E-17
hsa-miR-625-3p	-1.9712322	5.95654641	6.62E-06	1.54E-05
hsa-miR-15b-3p	-1.9824337	8.42095627	1.22E-16	5.37E-16
hsa-miR-32-3p	-2.0233815	5.11829609	0.0013281	0.0024057
hsa-miR-16-2-3p	-2.0594565	9.2687379	7.19E-20	3.67E-19
hsa-miR-374a-3p	-2.0614167	6.35074991	4.92E-08	1.38E-07
hsa-miR-133a-3p	-2.0772374	4.73779695	0.0070731	0.0119264
hsa-miR-1	-2.0781333	7.0234489	2.33E-12	8.68E-12
hsa-miR-7641	-2.1735726	11.2958233	2.67E-18	1.26E-17
hsa-miR-26a-5p	-2.1814089	15.2968747	4.51E-39	4.77E-38
hsa-miR-548k	-2.2869856	5.45356698	5.86E-06	1.39E-05
hsa-let-7a-5p	-2.2977814	13.7860112	1.24E-49	1.97E-48
hsa-miR-218-5p	-2.3256167	7.07630373	1.09E-10	3.75E-10
hsa-miR-130b-5p	-2.3361136	6.90074204	1.64E-12	6.19E-12
hsa-miR-3609	-2.3483465	5.5078573	0.0004114	0.0008101
hsa-miR-135b-5p	-2.4480439	7.72360205	1.70E-16	7.40E-16
hsa-miR-1180-3p	-2.4639653	8.38849613	6.48E-24	4.11E-23
hsa-miR-505-3p	-2.4844886	7.01252124	5.91E-16	2.53E-15
hsa-miR-935	-2.5090269	5.61344913	3.41E-07	8.86E-07
hsa-let-7e-5p	-2.5656847	9.49831852	7.69E-30	6.25E-29
hsa-miR-28-3p	-2.586018	10.8975487	1.48E-38	1.47E-37
hsa-miR-663b	-2.7274469	4.50504049	0.0004535	0.0008873

hsa-miR-455-3p	-2.8519706	6.63237027	2.05E-14	8.57E-14
hsa-let-7i-5p	-2.888893	16.3611296	5.07E-70	1.61E-68
hsa-miR-328-3p	-2.902498	7.36785438	7.10E-22	3.88E-21
hsa-miR-28-5p	-2.928071	8.64232507	9.55E-35	8.41E-34
hsa-miR-143-3p	-2.9829542	9.67359029	5.68E-46	7.51E-45
hsa-let-7d-5p	-3.1684967	10.9039653	1.08E-61	2.45E-60
hsa-miR-1246	-3.3894718	14.7254882	5.10E-28	3.76E-27
hsa-miR-34c-5p	-3.403589	4.93835579	4.83E-05	0.0001063
hsa-miR-184	-3.4676688	4.41930027	0.0007333	0.0014088
hsa-miR-15b-5p	-3.562668	9.81716952	4.35E-40	4.92E-39
hsa-miR-451a	-3.631175	7.58304938	1.16E-17	5.25E-17
hsa-miR-1248	-3.7897823	6.86006916	1.39E-10	4.74E-10
hsa-miR-122-5p	-4.5505061	7.30769212	4.77E-24	3.08E-23
hsa-miR-548ah-3p	-4.5910664	5.18869385	1.57E-08	4.60E-08
hsa-miR-548p	-4.7520979	5.31293574	3.09E-09	9.69E-09
hsa-miR-146a-3p	-5.9961491	4.69750804	8.13E-07	2.01E-06
hsa-miR-181a-3p	-6.1983007	4.83722402	1.47E-07	3.96E-07
hsa-miR-551a	-6.2395963	4.86846099	2.81E-08	8.04E-08
hsa-miR-598-3p	-6.287747	4.90225145	5.91E-08	1.63E-07
hsa-miR-155-5p	-9.1046525	9.22715749	6.63E-95	3.00E-93
hsa-miR-146a-5p	-12.229015	13.097146	1.11E-239	3.52E-237

Supplementary Table S3. Differentially expressed exosomal miRNAs were detected by exosomal miRNA sequencing in the supernatant of highly liver metastatic potential gastric cancer cell line MKN45-HL compared with its parental cell line MKN45 (FoldChange⩾2 or ⩽0.5; FDRp value < 0.05).
