# Supplementary material for: Gastric cancer-derived exosomal miR-519a-3p promotes liver metastasis by inducing intrahepatic M2-like macrophage-mediated angiogenesis
Source: J Exp Clin Cancer Res. 2022 Oct 10;41:296. doi: 10.1186/s13046-022-02499-8 (PMC9549645; doi:10.1186/s13046-022-02499-8)
Supplement: Supplementary file 3 — Additional file 3. [file 13046_2022_2499_MOESM3_ESM.pdf]

Gene ID	Gene Symbol	log2FC	PValue
100529144	CORO7-PAM16	-21.62425861	3.18E-08
106865373	GET1-SH3BGR	-5.814862063	0.132951906
632	BGLAP	-5.314336629	2.75E-04
110384692	LOC110384692	-5.106122962	0.192800949
24150	TP53TG3	-4.669133389	0.184997694
414059	TBC1D3B	-4.032501512	0.036726297
102723547	CSAG2	-4.028104301	0.306143437
111188157	LYNX1-SLURP2	-4.027360311	0.027630066
552891	DNAJC25-GNG10	-3.858173769	0.327449084
2268	FGR	-3.857224435	0.037684747
105375816	LOC105375816	-3.856538019	0.040529146
474382	H2AB1	-3.660017118	0.06219603
84623	KIRREL3	-3.446296944	0.111456906
127550	A3GALT2	-3.445209315	0.096608325
157807	CLVS1	-3.442936888	0.092511972
8366	H4C2	-3.44089043	0.089712422
107080644	CNPY3-GNMT	-3.390079324	2.91E-04
731220	RFX8	-3.213734828	0.060286626
727909	GOLGA8Q	-3.187099563	0.203919863
285588	EFCAB9	-3.181284709	0.14541234
140290	TCP10L	-3.179864893	0.20575912
84218	TBC1D3F	-3.179864893	0.20575912
195977	ANTXRL	-3.178859373	0.167617133
6277	S100A6	-3.175297015	0.001521228
2041	EPHA1	-3.174921567	0.16854524
105371191	LOC105371191	-3.174687842	0.168601425
284366	KLK9	-3.172134453	0.169204901
102724127	TP53TG3F	-3.166526087	0.209171585
1009	CDH11	-2.902040909	0.132262277
100505841	LOC100505841	-2.90047399	0.132661813
30010	NXPH1	-2.897669119	0.105747359
266812	NAP1L5	-2.895585428	0.123385167
3892	KRT86	-2.894578049	0.103094825
1586	CYP17A1	-2.889836813	0.123365552
4607	MYBPC3	-2.861292541	0.249215304
151790	WDR49	-2.858054967	0.222907975
57626	KLHL1	-2.858054967	0.222907975
105377641	LOC105377641	-2.857924294	0.291282087
199675	MCEMP1	-2.857924294	0.291282087
373856	USP41	-2.857924294	0.291282087
286234	SPATA31E1	-2.854967582	0.251047806
100288520	USP17L15	-2.854671289	0.255179607
23220	DTX4	-2.853332543	0.224215736
375686	SPATC1	-2.853332543	0.224215736
148170	CDC42EP5	-2.851503983	0.252055902
1907	EDN2	-2.851503983	0.252055902
389690	MROH5	-2.851503983	0.252055902
56676	ASCL3	-2.851503983	0.252055902
116	ADCYAP1	-2.850032494	0.256525304
101928917	HSFX3	-2.711961287	0.49173351
79730	NSUN7	-2.708128662	0.16403576
1278	COL1A2	-2.706235187	0.150070782
2925	GRPR	-2.705251464	0.14405598
440050	KRTAP5-7	-2.705251464	0.14405598
645432	ARRDC5	-2.70342186	0.144282732

7053	TGM3	-2.701597651	0.172694735
445582	POTEE	-2.698100217	0.163323958
4481	MSR1	-2.697463779	0.145647359
345557	PLCXD3	-2.695696904	0.152110384
66037	BOLL	-2.587210528	0.034387937
259294	TAS2R19	-2.586342608	0.078339798
646817	SETSIP	-2.484221321	0.215355241
105375107	LOC105375107	-2.480150126	0.195378526
123264	SLC51B	-2.480149743	0.195221841
389336	C5orf46	-2.479986669	0.201095705
57419	SLC24A3	-2.478042356	0.195707677
441168	CALHM6	-2.476106071	0.196382994
143662	MUC15	-2.450013511	0.413613571
56140	PCDHA8	-2.450013511	0.413613571
5790	PTPRCAP	-2.450013511	0.413613571
728911	CT45A2	-2.450013511	0.413613571
100507203	SMLR1	-2.447383139	0.376834872
259293	TAS2R30	-2.447383139	0.376834872
390664	C1QTNF8	-2.447383139	0.376834872
926	CD8B	-2.447383139	0.376834872
94160	ABCC12	-2.447383139	0.376834872
400823	FAM177B	-2.445093728	0.377594142
4489	MT1A	-2.445093728	0.377594142
5919	RARRES2	-2.445093728	0.377594142
388646	GBP7	-2.443185395	0.378225445
388649	C1orf146	-2.443185395	0.378225445
4493	MT1E	-2.443185395	0.378225445
7010	TEK	-2.443185395	0.378225445
105372585	LOC105372585	-2.442873246	0.415956437
2904	GRIN2B	-2.442873246	0.415956437
5232	PGK2	-2.442873246	0.415956437
647060	SPATA31A1	-2.442873246	0.415956437
727905	SPATA31A5	-2.442873246	0.415956437
1414	CRYBB1	-2.44088942	0.347843086
196993	CT62	-2.44088942	0.347843086
339302	CPLX4	-2.44088942	0.347843086
346171	ZFP57	-2.44088942	0.347843086
3861	KRT14	-2.44088942	0.347843086
5507	PPP1R3C	-2.44088942	0.347843086
57467	HHATL	-2.44088942	0.347843086
80343	SEL1L2	-2.44088942	0.347843086
105375938	LOC105375938	-2.438589516	0.379751849
793	CALB1	-2.438589516	0.379751849
163589	TDRD5	-2.43667254	0.380387122
64446	DNAI2	-2.43667254	0.380387122
647286	RD3L	-2.43667254	0.380387122
105369535	LOC105369535	-2.434366001	0.381154276
386618	KCTD4	-2.434366001	0.381154276
388333	SPDYE4	-2.434366001	0.381154276
57093	TRIM49	-2.434366001	0.381154276
105372714	LOC105372714	-2.429704427	0.420278875
122042	RXFP2	-2.429704427	0.420278875
171484	FAM9C	-2.429704427	0.420278875
187	APLNR	-2.429704427	0.420278875
285641	SLC36A3	-2.429704427	0.420278875
50508	NOX3	-2.429704427	0.420278875

107986554	LOC107986554	-2.325220232	0.133706865
7478	WNT8A	-2.24930798	0.043784944
10803	CCR9	-2.21927392	0.271470092
680	BRS3	-2.21927392	0.271470092
255411	TEX49	-2.217004069	0.291562814
277	AMY1B	-2.215714702	0.268264065
342933	ZSCAN5B	-2.215714702	0.268264065
102724657	LOC102724657	-2.213088945	0.293033517
613211	DEFB134	-2.212144779	0.273323824
8653	DDX3Y	-2.212144401	0.273372421
100288072	SDR42E2	-2.212142799	0.273578562
4070	TACSTD2	-2.210693248	0.293530217
727684	C1orf195	-2.209535603	0.300168454
7224	TRPC5	-2.171869123	0.191031602
132200	C3orf49	-2.164614921	0.172341508
6447	SCG5	-2.160219889	0.236062703
100529215	ZNF559-ZNF177	-2.148943899	2.36E-04
150094	SIK1	-2.136156655	1.56E-11
653643	GOLGA6D	-2.118616139	0.124444677
441581	FRG2B	-2.112376569	0.154654359
259283	MDS2	-2.003043533	0.216653331
158833	AWAT1	-2.001090235	0.12688044
729747	ZNF878	-2.00021531	0.217446629
8784	TNFRSF18	-1.999381604	0.217230932
105375112	LOC105375112	-1.999371913	0.213456975
5696	PSMB8	-1.996193162	0.255624019
64499	TPSB2	-1.99469381	0.271440909
100132565	GOLGA8F	-1.959161702	2.80E-04
105371371	LOC105371371	-1.902748584	0.426178472
2213	FCGR2B	-1.902748584	0.426178472
101929738	LOC101929738	-1.901546898	0.115102721
400757	C1orf141	-1.899107799	0.392792982
283600	SLC25A47	-1.897536191	0.397586919
126755	LRRC38	-1.896097797	0.372830755
127254	ERICH3	-1.896096506	0.37251633
5544	PRB3	-1.896096506	0.37251633
389161	ANKUB1	-1.89601039	0.428130993
100133093	FAM25G	-1.89456111	0.373035594
102724652	CRYAA2	-1.89456111	0.373035594
79370	BCL2L14	-1.89456111	0.373035594
155051	CRYGN	-1.893244447	0.39484515
10223	GPA33	-1.891739801	0.374049629
121793	TEX29	-1.891739801	0.374049629
1411	CRYBA1	-1.891739801	0.374049629
6588	SLN	-1.891739801	0.374049629
1441	CSF3R	-1.891738561	0.37430561
5949	RBP3	-1.891738561	0.37430561
402117	VWC2L	-1.890038661	0.395934186
57687	VAT1L	-1.888661508	0.400516621
101059915	LOC101059915	-1.888660899	0.400582619
153643	FAM81B	-1.888658315	0.400862591
51237	MZB1	-1.887042707	0.401130398
64078	SLC28A3	-1.887039341	0.401410899
415	ARSL	-1.883595168	0.43227669
165721	DNAJB8	-1.883590805	0.43257565
4063	LY9	-1.868320434	0.168165591

634	CEACAM1	-1.866594258	0.178090151
105376714	LOC105376714	-1.864980486	0.594896749
219681	ARMC3	-1.864980486	0.594896749
256369	CCDC197	-1.864980486	0.594896749
29113	C6orf15	-1.864980486	0.594896749
6530	SLC6A2	-1.864980486	0.594896749
653192	TRIM43B	-1.864980486	0.594896749
6581	SLC22A3	-1.864980486	0.594896749
728049	CT47A8	-1.864980486	0.594896749
728090	CT47A2	-1.864980486	0.594896749
92129	RIPPLY1	-1.864980486	0.594896749
93034	NT5C1B	-1.864980486	0.594896749
1043	CD52	-1.861235915	0.565565187
105378161	LOC105378161	-1.861235915	0.565565187
116173	CMTM5	-1.861235915	0.565565187
164237	WFDC13	-1.861235915	0.565565187
1770	DNAH9	-1.861235915	0.565565187
1828	DSG1	-1.861235915	0.565565187
283677	REC114	-1.861235915	0.565565187
3158	HMGCS2	-1.861235915	0.565565187
344807	CD200R1L	-1.861235915	0.565565187
399939	TRIM49D1	-1.861235915	0.565565187
5265	SERPINA1	-1.861235915	0.565565187
5729	PTGDR	-1.861235915	0.565565187
5956	OPN1LW	-1.861235915	0.565565187
642623	UBTFL1	-1.861235915	0.565565187
8852	AKAP4	-1.861235915	0.565565187
91703	ACY3	-1.861235915	0.565565187
9256	TSPOAP1	-1.861235915	0.565565187
105375809	LOC105375809	-1.857897499	0.597135456
105379198	LOC105379198	-1.857897499	0.597135456
146862	UNC45B	-1.857897499	0.597135456
165904	XIRP1	-1.857897499	0.597135456
286887	KRT6C	-1.857897499	0.597135456
353132	LCE1B	-1.857897499	0.597135456
3561	IL2RG	-1.857897499	0.597135456
400830	DEFB132	-1.857897499	0.597135456
53831	GPR84	-1.857897499	0.597135456
91074	ANKRD30A	-1.857897499	0.597135456
101060588	LOC101060588	-1.854979327	0.567637968
112267910	LOC112267910	-1.854979327	0.567637968
1485	CTAG1B	-1.854979327	0.567637968
152940	C4orf45	-1.854979327	0.567637968
26526	TSPAN16	-1.854979327	0.567637968
3249	HPN	-1.854979327	0.567637968
3293	HSD17B3	-1.854979327	0.567637968
4622	MYH4	-1.854979327	0.567637968
50617	ATP6V0A4	-1.854979327	0.567637968
56143	PCDHA5	-1.854979327	0.567637968
613210	DEFB136	-1.854979327	0.567637968
6332	SCN7A	-1.854979327	0.567637968
64092	SAMSN1	-1.854979327	0.567637968
84873	ADGRG7	-1.854979327	0.567637968
85479	DNAJC5B	-1.854979327	0.567637968
100653515	CEP295NL	-1.85155339	0.568773807
129446	XIRP2	-1.85155339	0.568773807

145942	TMCO5A	-1.85155339	0.568773807
259286	TAS2R40	-1.85155339	0.568773807
259291	TAS2R45	-1.85155339	0.568773807
26693	OR2V1	-1.85155339	0.568773807
342574	KRT27	-1.85155339	0.568773807
386746	MRGPRG	-1.85155339	0.568773807
387837	CLEC12B	-1.85155339	0.568773807
392391	OR5C1	-1.85155339	0.568773807
494188	FBXO47	-1.85155339	0.568773807
6017	RLBP1	-1.85155339	0.568773807
6039	RNASE6	-1.85155339	0.568773807
645745	MT1HL1	-1.85155339	0.568773807
138307	LCN8	-1.844832028	0.601253336
140893	RBBP8NL	-1.844832028	0.601253336
260429	PRSS33	-1.844832028	0.601253336
340900	EBLN1	-1.844832028	0.601253336
3575	IL7R	-1.844832028	0.601253336
3598	IL13RA2	-1.844832028	0.601253336
442186	OR2J3	-1.844832028	0.601253336
51365	PLA1A	-1.844832028	0.601253336
653220	XAGE1A	-1.844832028	0.601253336
728269	MAGEA9B	-1.844832028	0.601253336
765	CA6	-1.844832028	0.601253336
83661	MS4A8	-1.844832028	0.601253336
137735	ABRA	-1.808325357	0.316685244
283897	C16orf54	-1.80659686	0.065058083
1015	CDH17	-1.804387616	0.274184681
51179	HAO2	-1.804387616	0.274184681
79674	VEPH1	-1.804258485	0.300688176
254528	MEIOB	-1.804133374	0.279071397
11211	FZD10	-1.801621952	0.296258567
4829	NMBR	-1.801621952	0.296258567
286749	STON1-GTF2A1L	-1.781221116	0.030833935
91227	GGTLC2	-1.762842523	0.158716662
29970	SCHIP1	-1.737479625	0.02247757
8477	GPR65	-1.733558705	0.223229563
90167	FRMD7	-1.731964954	0.214050317
343702	XKR7	-1.723664091	0.009781028
9547	CXCL14	-1.707115383	0.20184973
26683	OR4F3	-1.651094372	0.070363318
55001	TTC22	-1.612263976	0.008525884
161502	CFAP161	-1.592811131	0.292004599
10398	MYL9	-1.587446013	0.304174053
89876	CFAP91	-1.586519832	0.373952922
55106	SLFN12	-1.585835294	0.357996218
1236	CCR7	-1.584816562	0.198500945
164312	LRRN4	-1.58474857	0.354075099
400831	C20orf202	-1.584581616	0.15855318
128488	WFDC12	-1.583687796	0.263421272
5251	PHEX	-1.583403952	0.116393035
136263	SSMEM1	-1.582399914	0.374499386
3821	KLRC1	-1.582399914	0.374499386
6370	CCL25	-1.582297859	0.266121439
346	APOC4	-1.581094868	0.382309463
2938	GSTA1	-1.578684418	0.375642523
51703	ACSL5	-1.577209592	0.401520257

338440	ANO9	-1.521713082	0.083246758
729857	RGPD2	-1.510113605	0.169663197
416	ARSF	-1.497846897	0.151124842
151112	ZSWIM2	-1.48748424	0.562233061
285659	OR2V2	-1.485005737	0.537549988
339403	RXFP4	-1.485005737	0.537549988
57113	TRPC7	-1.485001923	0.53724097
10017	BCL2L10	-1.482921312	0.537981056
611	OPN1SW	-1.482921312	0.537981056
2849	GPR26	-1.482920729	0.537908086
107984043	LOC107984043	-1.481447746	0.199028853
653598	PPIAL4C	-1.481184275	0.538908196
728957	ZNF705D	-1.481184275	0.538908196
7512	XPNPEP2	-1.481184275	0.538908196
11181	TREH	-1.481182898	0.538598098
1811	SLC26A3	-1.481182898	0.538598098
343413	FCRL6	-1.481182898	0.538598098
719	C3AR1	-1.481182898	0.538598098
8284	KDM5D	-1.481182898	0.538598098
6097	RORC	-1.481182574	0.538525015
2259	FGF14	-1.480926989	0.564884533
4490	MT1B	-1.479096177	0.519347917
768	CA9	-1.479096177	0.519347917
80133	MROH9	-1.479096177	0.519347917
9365	KL	-1.479096177	0.519347917
284348	LYPD5	-1.479096153	0.519058106
344191	EVX2	-1.479096153	0.519058106
3880	KRT19	-1.479096153	0.519058106
56302	TRPV5	-1.479096153	0.519058106
22895	RPH3A	-1.479096148	0.518989811
4951	OCM2	-1.477010134	0.540009726
10578	GNLY	-1.477009817	0.540083092
112744	IL17F	-1.477008473	0.540394387
259292	TAS2R46	-1.477008473	0.540394387
4958	OMD	-1.477008473	0.540394387
643965	TMEM88B	-1.477008473	0.540394387
81697	OR2B2	-1.475265955	0.540629096
145645	TERB2	-1.475265376	0.540702578
4625	MYH7	-1.475265376	0.540702578
347169	OR1B1	-1.475262918	0.541014361
10744	PTTG2	-1.473173772	0.541373869
1301	COL11A1	-1.473173772	0.541373869
6756	SSX1	-1.473172867	0.541447492
3007	H1-3	-1.473169031	0.541759878
55363	HEMGN	-1.473169031	0.541759878
127064	OR2T12	-1.468833484	0.568631073
23017	FAIM2	-1.468833484	0.568631073
23624	CBLC	-1.468833484	0.568631073
100190949	C5orf52	-1.468832095	0.568708105
2266	FGG	-1.468832095	0.568708105
392517	NCBP2L	-1.468832095	0.568708105
9402	GRAP2	-1.468832095	0.568708105
154064	RAET1L	-1.468826209	0.569034918
58511	DNASE2B	-1.46592813	0.296532844
100996693	SPEGNB	-1.457647475	0.237341439
254158	CXorf58	-1.453854377	0.254090676

6511	SLC1A6	-1.453629433	0.270069955
90594	ZNF439	-1.438844755	0.130848104
197	AHSG	-1.424468159	0.372115791
8842	PROM1	-1.41807012	0.334578483
23504	RIMBP2	-1.411739553	0.334096674
100129515	ETDB	-1.411693427	0.40597116
1071	CETP	-1.40910616	0.342464412
145781	GCOM1	-1.361419193	0.295533569
102723502	LOC102723502	-1.350635144	0.077882392
342096	GOLGA6A	-1.338061366	0.054993675
497190	CLEC18B	-1.331216438	0.370353361
2642	GCGR	-1.327358409	0.479326541
347516	DGAT2L6	-1.326466505	0.480176094
5343	PLGLB1	-1.326124741	0.32484643
10242	KCNMB2	-1.325133391	0.315335538
6569	SLC34A1	-1.324050903	0.464727048
388381	C17orf98	-1.323657357	0.460904554
128209	KLF17	-1.322676973	0.480573601
401613	SERTM2	-1.322528696	0.465448179
728226	GGTLC3	-1.322343355	0.310991162
401258	RAB44	-1.322160457	0.295059247
253012	HEPACAM2	-1.321515723	0.461766787
7036	TFR2	-1.321515723	0.461766787
107986244	LOC107986244	-1.32018666	0.140820981
654429	LRTM2	-1.320161475	0.334600494
2565	GABRG1	-1.320113141	0.477434637
6276	S100A5	-1.319543922	0.481701132
6869	TACR1	-1.319343456	0.462062908
112267947	LOC112267947	-1.319033536	0.20137463
399968	PATE4	-1.318725911	0.148093251
3689	ITGB2	-1.318442639	0.315795828
79698	ZMAT4	-1.318136787	0.466628601
2113	ETS1	-1.317650085	0.216645376
390667	PTX4	-1.317071386	0.310996618
55784	MCTP2	-1.316952625	0.312844986
344387	CDKL4	-1.316032514	0.463398164
866	SERPINA6	-1.313255837	0.502124148
112714	TUBA3E	-1.311936168	0.253193477
1646	AKR1C2	-1.309691703	0.509623197
140825	NEURL2	-1.308577896	0.030781515
100861540	CYP3A7-CYP3A51P	-1.304091641	0.739108543
57047	PLSCR2	-1.288735466	0.13880782
8641	PCDHGB4	-1.273298352	0.039146898
343263	MYBPHL	-1.263808537	0.273157164
84871	AGBL4	-1.26332697	0.285789069
146433	IL34	-1.263109822	0.283850283
22829	NLGN4Y	-1.262162942	0.291187173
2863	GPR39	-1.251449826	0.262898849
84992	PIGY	-1.228124034	0.43685821
105376791	LOC105376791	-1.226276696	0.448440538
135886	TMEM270	-1.223202075	0.42340668
160762	CCDC63	-1.222480154	0.412500219
56135	PCDHAC1	-1.221402801	0.419100384
168400	DDX53	-1.22126641	0.248498858
676	BRDT	-1.221094592	0.110477379
2267	FGL1	-1.219689086	0.451020785

9407	TMPRSS11D	-1.218710205	0.420139238
151254	C2CD6	-1.217516049	0.456324687
57497	LRFN2	-1.191759421	0.24215116
93589	CACNA2D4	-1.191144368	0.234053903
101930420	LOC101930420	-1.189078905	0.230313864
3627	CXCL10	-1.17342855	0.393946257
338755	OR2AG2	-1.170978755	0.228446856
56106	PCDHGA10	-1.170709927	0.210301742
26085	KLK13	-1.170048409	0.377273289
3769	KCNJ13	-1.168198309	0.373310571
79849	PDZD3	-1.168198309	0.373310571
9615	GDA	-1.166440909	0.215302629
56	ACRV1	-1.156479179	0.135926722
92346	C1orf105	-1.133810726	0.34113983
9705	ST18	-1.133810726	0.34113983
100533178	PRORY	-1.130783266	0.372706433
51411	BIN2	-1.129189909	0.360558722
8909	ENDOU	-1.127446451	0.206699191
285231	FBXW12	-1.124106116	0.163260181
10864	SLC22A7	-1.103719251	0.150217016
283298	OLFML1	-1.099617577	0.276145929
4050	LTB	-1.098459992	0.286211965
107984449	LOC107984449	-1.097273875	0.301910379
8993	PGLYRP1	-1.095669157	0.274283777
102723382	LOC102723382	-1.091714326	0.195362774
441864	TARM1	-1.087741116	0.248658336
84063	KIRREL2	-1.085913868	0.252073916
163688	CALML6	-1.085370566	0.046726084
6753	SSTR3	-1.074337844	0.227146193
8797	TNFRSF10A	-1.068629856	0.204741167
221421	RSPH9	-1.064285212	0.074001496
100534592	URGCP-MRPS24	-1.063113985	0.515574025
645202	LOC645202	-1.061157808	0.286791069
3122	HLA-DRA	-1.046290799	0.146039005
54596	L1TD1	-1.025724197	0.077377844
202658	TRIM39-RPP21	-1.014574474	0.65649067
6523	SLC5A1	-1.005064404	0.492520259
57282	SLC4A10	-1.004658681	0.429698884
3827	KNG1	-1.00436325	0.490194005
125704	DIPK1C	-1.00390227	0.554106724
101060017	LOC101060017	-1.003832601	0.38805439
23040	MYT1L	-1.003564693	0.526345476
653361	NCF1	-1.003132517	0.480011959
5126	PCSK2	-1.002648069	0.614211374
5294	PIK3CG	-1.002648069	0.614211374
563	AZGP1	-1.002648069	0.614211374
85445	CNTNAP4	-1.001967626	0.651139394
440955	TMEM89	-1.001824859	0.533166215
100652833	LOC100652833	-1.001665435	0.49520091
2147	F2	-1.001618365	0.463545502
8351	H3C4	-1.001505757	0.299931528
5444	PON1	-1.001083016	0.634964937
11170	FAM107A	-1.001078621	0.467374427
123876	ACSM2A	-1.000860946	0.514859048
425054	VCX3B	-1.00082582	0.626772302
6361	CCL17	-1.00057146	0.162419858

142685	ASB15	-1.000356794	0.630074304
3624	INHBA	-1.000116224	0.251867622
158401	SHOC1	1.000772148	0.634729162
29943	PADI1	1.000772148	0.634729162
219578	ZNF804B	1.000811781	0.49070096
116512	MRGPRD	1.000830327	0.527190348
10232	MSLN	1.000903731	0.407023446
8335	H2AC4	1.001154719	0.52748994
10077	TSPAN32	1.001212569	0.406679934
26191	PTPN22	1.00125459	0.218086513
3081	HGD	1.001362505	0.54528748
339766	MROH2A	1.001422926	0.514740577
10568	SLC34A2	1.001556667	0.614317045
119395	CALHM3	1.001556667	0.614317045
147744	TMEM190	1.001559871	0.527301395
54825	CDHR2	1.001582873	0.389135809
6372	CXCL6	1.001936876	0.117771789
4232	MEST	1.002075744	0.406369257
383	ARG1	1.002307563	0.401741136
6445	SGCG	1.002755742	0.32524415
159989	DEUP1	1.003048647	0.597046497
4360	MRC1	1.003107573	0.519979688
128025	WDR64	1.00325573	0.327549079
2911	GRM1	1.003292881	0.321477955
1621	DBH	1.003297124	0.575673663
375759	C9orf50	1.003340005	0.204550963
645073	GAGE12G	1.003367064	0.610956717
376132	LRRC10	1.003437648	0.29112376
4110	MAGEA11	1.003640444	0.596796758
105369246	LOC105369246	1.003838546	0.378368395
940	CD28	1.003929444	0.325941287
8404	SPARCL1	1.003978308	0.362527035
29986	SLC39A2	1.004111823	0.454136416
57471	ERMN	1.004162983	0.567052197
83998	REG4	1.004355915	0.462335786
8612	PLPP2	1.004385288	0.451533962
90134	KCNH7	1.005003408	0.525700867
408263	FNDC9	1.005170319	0.596395195
283971	CLEC18C	1.005201504	0.628624317
144501	KRT80	1.00635515	0.609473086
1393	CRHBP	1.006671318	0.612937049
169044	COL22A1	1.006671318	0.612937049
1634	DCN	1.006918549	0.609959786
109729126	FAM236C	1.007954563	0.625129148
338442	HCAR2	1.008986545	0.488205874
259290	TAS2R31	1.010161686	0.608624436
5657	PRTN3	1.04707989	0.0291464
79825	EFCC1	1.060763774	0.169636738
5650	KLK7	1.067857679	0.218814444
956	ENTPD3	1.069210027	0.212542909
3008	H1-4	1.069710115	0.200868175
10148	EBI3	1.070378535	0.219474978
2203	FBP1	1.072345401	0.206736564
105377022	LOC105377022	1.085144569	0.275495489
5913	RAPSN	1.100463743	0.276767468
3834	KIF25	1.100762876	0.272035577

283316	CD163L1	1.102125566	0.281136501
392490	FLJ44635	1.105735741	0.137746815
107987477	LOC107987477	1.111008822	0.452118616
29974	A1CF	1.117107166	0.152519617
5731	PTGER1	1.12720527	0.156774193
84870	RSPO3	1.138350846	0.177937252
200008	CDCP2	1.138846321	0.175474122
246176	GAS2L2	1.139930832	0.172228679
84684	INSM2	1.144509974	0.19658366
440804	RIMBP3B	1.145575702	0.431310317
1264	CNN1	1.147851072	0.109543299
1394	CRHR1	1.151167109	0.20320584
729873	TBC1D3	1.162614255	0.0053499
102724646	LOC102724646	1.162664037	0.154197513
6441	SFTPD	1.168451605	0.377925444
149628	PYHIN1	1.171224347	0.251983591
23650	TRIM29	1.172213436	0.220274756
101928436	LOC101928436	1.174230886	0.370892747
105372319	LOC105372319	1.175336217	0.383590051
26025	PCDHGA12	1.182023093	0.411912481
80125	CCDC33	1.191566267	0.262853285
112268102	LOC112268102	1.191801997	0.263991403
730112	FAM166B	1.1924977	0.241798097
107984345	SMIM38	1.220954567	0.272241306
3698	ITIH2	1.221540065	0.261776624
399967	PATE2	1.222035038	0.423534757
149297	FAM78B	1.222159468	0.109214963
353149	TBC1D26	1.222279297	0.115608775
282763	OR51B5	1.223121852	0.244521464
140458	ASB5	1.223354907	0.438014545
114784	CSMD2	1.224024724	0.242784053
121006	FAM186A	1.22447661	0.417997664
389073	C2orf80	1.2256495	0.444588432
101929726	MYMX	1.225766852	0.432838072
3013	H2AC7	1.226074495	0.448052597
254956	MORN5	1.226709114	0.275483138
105378950	LOC105378950	1.227476602	0.416828576
135656	MUCL3	1.229345966	0.456576075
414235	PRR26	1.246163644	0.221901612
100529251	CKLF-CMTM1	1.248247695	0.197540579
101929469	TEX52	1.250540143	0.185341408
54738	FEV	1.251987465	0.193740753
129807	NEU4	1.262787305	0.273153498
344758	GPR149	1.271333709	0.301669494
102723360	LOC102723360	1.285158002	0.106601244
3960	LGALS4	1.316837183	0.338752351
388419	BTBD17	1.317499319	0.46699417
7293	TNFRSF4	1.317682121	0.482665141
6340	SCNN1G	1.318568876	0.500563709
27319	BHLHE22	1.318832492	0.48220842
388743	CAPN8	1.319379355	0.481713684
2928	GSC2	1.320144347	0.461851957
5651	TMPRSS15	1.320463708	0.314973911
1996	ELAVL4	1.320580831	0.312040308
8689	KRT36	1.321507966	0.327702382
221823	PRPS1L1	1.32168212	0.465316432

653489	RGPD3	1.321776541	0.006234423
837	CASP4	1.32195838	0.297653129
54937	SOHLH2	1.322212464	0.461018804
144423	GLT1D1	1.322835375	0.321525377
2793	GNGT2	1.323281073	0.461074768
348158	ACSM2B	1.323281073	0.461074768
54979	PLAAT2	1.323753112	0.481045709
2847	MCHR1	1.324296935	0.480550405
203328	SUSD3	1.32466753	0.480170206
389549	FEZF1	1.324960464	0.202256782
131578	LRRC15	1.325247746	0.460548446
154197	PNLDC1	1.325401775	0.326249309
58985	IL22RA1	1.325567173	0.305262986
101928268	LOC101928268	1.325834455	0.460046864
127731	VWA5B1	1.325834455	0.460046864
4583	MUC2	1.325834455	0.460046864
29122	PRSS50	1.32635096	0.463933017
441519	CT45A3	1.326894961	0.50719413
6286	S100P	1.331516587	0.478483526
4056	LTC4S	1.363940215	0.15506233
29126	CD274	1.371263454	0.064710383
9075	CLDN2	1.377017047	0.231120321
733	C8G	1.379811126	0.036845071
56107	PCDHGA9	1.382712257	0.220206545
7730	ZNF177	1.383090974	0.01271613
2207	FCER1G	1.412869269	0.381942168
3270	HRC	1.413576277	0.331980971
4969	OGN	1.41489341	0.332886424
151651	EFHB	1.415799232	0.099540471
388112	NANOGP8	1.416621943	0.215866446
1588	CYP19A1	1.418374316	0.339495731
401546	C9orf152	1.419483125	0.333962595
10346	TRIM22	1.419873657	0.342856097
55584	CHRNA9	1.419934974	0.363887213
11277	TREX1	1.42808924	0.389079672
197135	PATL2	1.429724361	0.042812418
28232	SLCO3A1	1.458077662	0.251679971
5552	SRGN	1.460750759	0.236265747
54681	P4HTM	1.461099706	0.240653205
83894	TTC29	1.47813106	0.540256387
84700	MYO18B	1.47813106	0.540256387
149647	FAM71A	1.478134127	0.539933363
121275	OR10AD1	1.478135804	0.539756894
222826	FAM217A	1.478135804	0.539756894
4656	MYOG	1.479002931	0.53994603
6512	SLC1A7	1.479005405	0.539623266
2701	GJA4	1.479006759	0.539446939
100129216	DEFB131B	1.481833752	0.538939567
80834	TAS1R2	1.481833752	0.538939567
131177	FAM3D	1.481834336	0.538617637
81551	STMN4	1.481834336	0.538617637
100329135	TRPC5OS	1.481834655	0.538441765
115749	C12orf56	1.482703446	0.517835183
22918	CD93	1.482703446	0.517835183
26083	TBC1D29P	1.482703446	0.517835183
388125	C2CD4B	1.482703446	0.517835183

4586	MUC5AC	1.482703446	0.517835183
3697	ITIH1	1.482703449	0.517999061
387733	IFITM5	1.482703449	0.517999061
55816	DOK5	1.482703449	0.517999061
100533183	ZNF664-RFLNA	1.482703455	0.518299077
139065	SLITRK4	1.482703455	0.518299077
146	ADRA1D	1.482703455	0.518299077
11131	CAPN11	1.483572357	0.537823564
28954	REM1	1.483573265	0.538320567
1581	CYP7A1	1.485670269	0.563512254
23708	GSPT2	1.486392717	0.536821454
6285	S100B	1.486392717	0.536821454
10911	UTS2	1.486396513	0.537317177
220082	CBY2	1.486396513	0.537317177
100288485	MTRNR2L7	1.487259477	0.536513207
5918	RARRES1	1.487259477	0.536513207
8600	TNFSF11	1.487259477	0.536513207
10107	TRIM10	1.487261135	0.536688203
23562	CLDN14	1.487261135	0.536688203
440533	PSG8	1.487264168	0.537008531
55891	LENEP	1.487264168	0.537008531
171558	PTCRA	1.488402095	0.562238749
8356	H3C12	1.502765329	0.167910774
728361	OVOL3	1.50373664	0.141658014
9496	TBX4	1.524726022	0.079362159
51412	ACTL6B	1.525536863	0.020365422
56138	PCDHA11	1.578601303	0.418302255
91734	IDI2	1.579486488	0.303146003
339512	CCDC190	1.580813352	0.40071
100132202	LOC100132202	1.582076155	0.407752685
438	ASMT	1.582114841	0.359004173
7201	TRHR	1.582408791	0.28921538
10993	SDS	1.582753321	0.397826335
3004	GZMM	1.584100092	0.284731394
114902	C1QTNF5	1.584417175	0.262495179
110806280	CCDC194	1.584498875	0.210287098
92086	GGTLC1	1.585119218	0.358374588
102724631	POTEB3	1.585707404	0.233177918
27296	TP53TG5	1.586452881	0.073019339
5225	PGC	1.586820958	0.387620011
6347	CCL2	1.586974398	0.357952124
200634	KRTCAP3	1.587269237	0.204130944
100529257	SYNJ2BP-COX16	1.588926082	0.094997672
57830	KRTAP5-8	1.588968524	0.414017293
84631	SLITRK2	1.591461868	0.376350522
56099	PCDHGB7	1.655607287	0.079744521
727830	SPATA31A3	1.671313505	0.162001319
353174	ZACN	1.680999928	0.123604364
27129	HSPB7	1.701469199	0.166410145
148198	ZNF98	1.707576023	0.206848251
6019	RLN2	1.719842032	0.434030181
1645	AKR1C1	1.729795257	0.242368047
3135	HLA-G	1.730200228	0.237133966
219770	GJD4	1.739144805	0.212216741
668	FOXL2	1.764814917	0.10340027
26659	OR7A5	1.806457542	0.303856668

56000	NXF3	1.806608761	0.280537816
92292	GLYATL1	1.80753154	0.279806396
2357	FPR1	1.808765492	0.31987143
5349	FXYD3	1.810790773	0.279182041
646457	C19orf67	1.810903076	0.120793388
5017	OVOL1	1.81164084	0.135010343
24141	LAMP5	1.812286972	0.294111442
219348	PLAC9	1.81233431	0.278682856
338376	IFNE	1.817815246	0.292295317
137970	UNC5D	1.850118753	0.599836446
170825	GSX2	1.850118753	0.599836446
26103	LRIT1	1.850118753	0.599836446
26246	OR2L2	1.850118753	0.599836446
2652	OPN1MW	1.850118753	0.599836446
3250	HPR	1.850118753	0.599836446
57818	G6PC2	1.850118753	0.599836446
9086	EIF1AY	1.850118753	0.599836446
10637	LEFTY1	1.850252769	0.084320866
100131303	DPEP2NB	1.856553173	0.567383586
107987238	LOC107987238	1.856553173	0.567383586
114769	CARD16	1.856553173	0.567383586
116369	SLC26A8	1.856553173	0.567383586
117245	PLAAT5	1.856553173	0.567383586
1472	CST4	1.856553173	0.567383586
2328	FMO3	1.856553173	0.567383586
2557	GABRA4	1.856553173	0.567383586
374860	ANKRD30B	1.856553173	0.567383586
4648	MYO7B	1.856553173	0.567383586
474354	LRRC18	1.856553173	0.567383586
480	ATP1A4	1.856553173	0.567383586
4990	SIX6	1.856553173	0.567383586
5340	PLG	1.856553173	0.567383586
5539	PPY	1.856553173	0.567383586
5948	RBP2	1.856553173	0.567383586
6010	RHO	1.856553173	0.567383586
7140	TNNT3	1.856553173	0.567383586
83795	KCNK16	1.856553173	0.567383586
100533181	FXYD6-FXYD2	1.85797954	0.566910528
10720	UGT2B11	1.85797954	0.566910528
119587	CPXM2	1.85797954	0.566910528
124538	OR4D2	1.85797954	0.566910528
140258	KRTAP13-1	1.85797954	0.566910528
155038	GIMAP8	1.85797954	0.566910528
171169	SPACA4	1.85797954	0.566910528
2191	FAP	1.85797954	0.566910528
219968	OR5B21	1.85797954	0.566910528
246744	STH	1.85797954	0.566910528
26298	EHF	1.85797954	0.566910528
286514	MAGEB18	1.85797954	0.566910528
3048	HBG2	1.85797954	0.566910528
3467	IFNW1	1.85797954	0.566910528
362	AQP5	1.85797954	0.566910528
50964	SOST	1.85797954	0.566910528
51059	FAM135B	1.85797954	0.566910528
56344	CABP5	1.85797954	0.566910528
5967	REG1A	1.85797954	0.566910528

7069	THRSP	1.85797954	0.566910528
7984	ARHGEF5	1.85797954	0.566910528
79843	FAM124B	1.85797954	0.566910528
8190	MIA	1.85797954	0.566910528
84072	HORMAD1	1.85797954	0.566910528
8735	MYH13	1.85797954	0.566910528
100287226	ZNF729	1.862716812	0.595862083
10143	CLEC3A	1.862716812	0.595862083
107987289	LOC107987289	1.862716812	0.595862083
116179	TGM7	1.862716812	0.595862083
161931	ADAD2	1.862716812	0.595862083
1758	DMP1	1.862716812	0.595862083
2248	FGF3	1.862716812	0.595862083
282616	IFNL2	1.862716812	0.595862083
390063	OR51I1	1.862716812	0.595862083
390616	ANKRD34C	1.862716812	0.595862083
53836	GPR87	1.862716812	0.595862083
5540	NPY4R	1.862716812	0.595862083
57172	CAMK1G	1.862716812	0.595862083
654231	OCM	1.862716812	0.595862083
107985022	LOC107985022	1.864020859	0.56490807
116441	TM4SF18	1.864020859	0.56490807
1230	CCR1	1.864020859	0.56490807
131377	KLHL40	1.864020859	0.56490807
200726	FAM237A	1.864020859	0.56490807
3443	IFNA6	1.864020859	0.56490807
3577	CXCR1	1.864020859	0.56490807
496	ATP4B	1.864020859	0.56490807
50863	NTM	1.864020859	0.56490807
51802	ASIC5	1.864020859	0.56490807
5739	PTGIR	1.864020859	0.56490807
6373	CXCL11	1.864020859	0.56490807
6759	SSX4	1.864020859	0.56490807
84647	PLA2G12B	1.864020859	0.56490807
121256	TMEM132D	1.865668339	0.594928918
1232	CCR3	1.865668339	0.594928918
127707	KLHDC7A	1.865668339	0.594928918
170371	TMEM273	1.865668339	0.594928918
2352	FOLR3	1.865668339	0.594928918
285093	RTP5	1.865668339	0.594928918
337968	KRTAP6-3	1.865668339	0.594928918
340543	TCEAL5	1.865668339	0.594928918
390442	OR11H4	1.865668339	0.594928918
7044	LEFTY2	1.865668339	0.594928918
7412	VCAM1	1.865668339	0.594928918
83897	KRTAP3-2	1.865668339	0.594928918
1297	COL9A1	1.883868606	0.18485258
11262	SP140	1.888813582	0.430613558
2844	GPR21	1.892754762	0.399447927
727837	SSX2B	1.894960428	0.394805188
100130933	SMIM6	1.894961842	0.394514452
100507537	STRIT1	1.894961842	0.394514452
402381	SOHLH1	1.895713497	0.372805468
7173	TPO	1.896296138	0.394351594
56112	PCDHGA3	1.896297265	0.39390271
1381	CRABP1	1.898435051	0.372030308

1804	DPP6	1.899074368	0.371670256
4345	CD200	1.899074368	0.371670256
1844	DUSP2	1.899074765	0.037181412
388960	C2orf78	1.899074765	0.371814116
80117	ARL14	1.901960413	0.392432194
7200	TRH	1.903591168	0.425850879
9363	RAB33A	1.930068629	0.061284547
26873	OPLAH	1.997253005	0.213751322
200232	FAM209A	2.004456482	0.137344925
219539	YPEL4	2.0054837	0.137234036
941	CD80	2.007042165	0.219079646
728378	POTEF	2.043351796	0.528589951
10750	GRAP	2.086846706	0.073857532
93649	MYOCD	2.173430053	0.163849684
7143	TNR	2.178155176	0.187598518
389941	C1QL3	2.205164646	0.027639268
345062	PRSS48	2.213300736	0.299538199
1369	CPN1	2.213851772	0.299370701
284654	RSPO1	2.216351191	0.272220745
84290	CAPNS2	2.218587713	0.267632479
22917	ZP1	2.218588186	0.267421138
124925	SEZ6	2.218588445	0.267305643
54714	CNGB3	2.219107643	0.267152467
284099	C17orf78	2.219625278	0.291281262
79747	ADGB	2.220804372	0.271022577
111064647	CSNKA2IP	2.221312816	0.266503206
2949	GSTM5	2.222502979	0.296510457
125958	OR7D4	2.223188122	0.296540109
81931	ZNF93	2.223692381	0.289816725
5314	PKHD1	2.224831013	0.296042703
337972	KRTAP19-5	2.32465687	0.13535306
105371045	PERCC1	2.435011564	0.418816771
343	AQP8	2.435011564	0.418816771
10249	GLYAT	2.439458621	0.379750245
135644	TRIM40	2.439458621	0.379750245
200403	VWA3B	2.439458621	0.379750245
2845	GPR22	2.439458621	0.379750245
79097	TRIM48	2.439458621	0.379750245
102723930	LOC102723930	2.440418655	0.379431187
1791	DNTT	2.440418655	0.379431187
100129407	FAM236A	2.444490184	0.346942812
147746	HIPK4	2.444490184	0.346942812
644100	ARL14EPL	2.444490184	0.346942812
79290	OR13A1	2.444490184	0.346942812
10022	INSL5	2.445444842	0.377763582
10871	CD300C	2.445444842	0.377763582
196335	OR56B4	2.445444842	0.377763582
4114	MAGEB3	2.445444842	0.377763582
6011	GRK1	2.445444842	0.377763582
1834	DSPP	2.44854619	0.376736292
29114	TAGLN3	2.44854619	0.376736292
5535	PPP3R2	2.44854619	0.376736292
326340	ZAR1	2.449500179	0.376420021
3512	JCHAIN	2.449500179	0.376420021
80168	MOGAT2	2.449500179	0.376420021
8290	H3-4	2.449500179	0.376420021

101929601	LOC101929601	2.450684974	0.413673855
282617	IFNL3	2.450684974	0.413673855
80201	HKDC1	2.450684974	0.413673855
51617	NSG2	2.480399155	0.195326621
105378947	LOC105378947	2.482704799	0.190911719
254050	LRRC43	2.483143652	0.194558541
6338	SCNN1B	2.484569925	0.194456414
5009	OTC	2.486603324	0.216564606
377630	USP17L2	2.48704898	0.214808082
5350	PLN	2.585297042	0.081796546
349334	FOXD4L4	2.699641021	0.165906219
29850	TRPM5	2.704274993	0.142500067
100526760	ABHD14A-ACY1	2.707115732	0.003130109
3316	HSPB2	2.853432371	0.25578985
79697	RIOX1	2.853432371	0.25578985
5313	PKLR	2.856527734	0.250848795
146167	SLC38A8	2.857331513	0.223348379
2307	FOXS1	2.857331513	0.223348379
11026	LILRA3	2.860281253	0.22253279
117579	RLN3	2.860281253	0.22253279
146849	CCDC42	2.860281253	0.22253279
2350	FOLR2	2.860281253	0.22253279
4161	MC5R	2.860974806	0.222341045
6846	XCL2	2.863377232	0.252925729
9934	P2RY14	2.901827977	0.122369282
389090	OR6B2	3.072284397	0.092467663
102724101	TP53TG3E	3.177212004	0.168205241
284257	BOD1L2	3.177212004	0.168205241
55998	NXF5	3.180301858	0.142086965
1440	CSF3	3.181411088	0.167216346
401387	LRRD1	3.183217958	0.141461001
100528032	KLRC4-KLRK1	3.184722492	0.204737102
391123	VSIG8	3.184722492	0.204737102
2939	GSTA2	3.18574028	0.166201616
653550	TP53TG3C	3.186317167	0.166066532
127435	PODN	3.21996022	0.054720772
79008	SLX1B	3.243689425	0.231831841
5624	PROC	3.355868262	0.042292506
2899	GRIK3	3.360085507	0.045491985
11036	GTF2A1L	3.434842167	0.148824461
2001	ELF5	3.446934847	0.091997207
57633	LRRN1	3.448605183	0.112643716
692094	MSMP	3.45080869	0.145474697
474381	H2AB2	3.666023128	0.06044118
83740	H2AB3	3.666023128	0.06044118
129868	TRIM43	3.666504156	0.078165533
729355	TP53TG3B	3.673971754	0.351631231
55281	TMEM140	4.433348362	0.25917336
27232	GNMT	4.856443785	0.172764073
407977	TNFSF12-TNFSF13	4.954236927	0.206557176
126961	H3C14	5.386835908	0.169196097
553158	PRR5-ARHGAP8	5.662912409	0.133708837
110354863	ZNF660-ZNF197	6.036750161	0.122999941
107987373	LOC107987373	6.356794096	0.104231463
284040	CDRT4	6.91784557	0.076905762

Supplementary Table S4. Differentially expressed mRNAs were detected by mRNA sequencing in PMA-treated THP-1 cells with knockdown of miR-519a-3p (log2FoldChange ⩾ 1 or ⩽ -1)
